# Fluoroacetonitrile‑Based Gel Polymer Electrolytes for Fast‑Charging Lithium Metal Batteries

**DOI:** 10.1002/advs.75528

**Published:** 2026-05-06

**Authors:** Zuoxin Yang, Jinyuan Wang, Zhe Tian, Boyi Song, Fangyi Cheng, Wangqing Zhang

**Affiliations:** ^1^ Institute of Polymer Chemistry College of Chemistry Key Laboratory of Functional Polymer Materials of the Ministry of Education Nankai University Tianjin China; ^2^ College of Chemistry Frontiers Science Center for New Organic Matter Nankai University Tianjin China; ^3^ College of Chemistry Key Laboratory of Advanced Energy Materials Chemistry of the Ministry of Education Nankai University Tianjin China; ^4^ Tianjin Key Laboratory of Functional Polymer Materials Nankai University Tianjin China

**Keywords:** fast‑charging, fluoroacetonitrile, gel polymer electrolytes, lithium metal batteries, solid electrolyte interfaces

## Abstract

Gel polymer electrolytes (GPEs) are promising electrolyte candidates for lithium metal batteries (LMBs) due to their favorable interfacial contact and enhanced safety. However, GPE electrolytes suffer from severe interfacial instability against lithium metal anodes (LMAs) and limited lithium‑ion transference number (*t*_Li+_), which significantly restricts the fast‑charging performance of LMBs. Herein, we propose a fast‑charging gel polymer electrolyte (GPE‑FN) using fluoroacetonitrile (FAN) as the solvent, with fluoroethylene carbonate (FEC) and LiNO_3_ as reduction additives. The GPE‑FN electrolyte addresses the interfacial incompatibility issues of FAN in LMBs by forming a stable solid electrolyte interphase (SEI) layer through the reduction of FEC and LiNO_3_ on LMA, which effectively prevents the continuous consumption of FAN. Furthermore, this SEI layer also effectively enhances the interfacial Li^+^ transport. The results show that the GPE‑FN electrolyte demonstrates a high ionic conductivity (σ) of 1.66 mS cm^−1^ and a high *t*_Li+_ of 0.848 at 25°C. Both LiFePO_4_ (LFP)||Li and LiNi_0.8_Co_0.1_Mn_0.1_O_2_ (NCM811)||Li cells based on the GPE‑FN electrolyte achieve 4100 cycles with 80.3% capacity retention and 800 cycles with 79.5% capacity retention at 5C, respectively, exhibiting excellent fast‑charging performance. Furthermore, full cells incorporating high‑loading cathodes demonstrate stable cycling, highlighting the promising potential of the GPE‑FN electrolyte.

## Graphical abstract

A gel polymer electrolyte using fluoroacetonitrile (FAN) as the solvent and fluoroethylene carbonate (FEC) and LiNO_3_ as additives is proposed for fast‐charging lithium metal batteries. These additives are preferentially reduced on the lithium metal anode surface to form a stable SEI layer, effectively suppressing the reductive decomposition of FAN. As a result, the battery based on this electrolyte achieves excellent cycling stability at 5C.

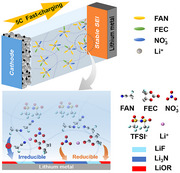

## 1 Introduction

The rapid advance of energy storage grids, electric vehicles, and consumer electronics arouses an urgent need for lithium‑ion secondary batteries with high energy density [1, 2, 3]. The lithium metal anode (LMA), owing to its high theoretical specific capacity and low redox potential, is regarded as one of the most promising alternatives for next‑generation high‑energy‑density batteries [4, 5, 6]. However, LMA is intrinsically incompatible with conventional liquid electrolytes, and leads to uncontrolled lithium dendrite growth during cycling [7, 8, 9, 10]. This dendrite not only accelerates capacity decaying, but even induces internal short circuit and severe hazard [11, 12, 13]. Compared with liquid electrolytes, solid‑state electrolytes have high mechanical strength, which can effectively resist lithium dendrite penetration, thereby significantly enhancing battery safety [14, 15]. However, the rigid nature of solid‑state electrolytes leads to high interfacial impedance owing to limited interfacial contact with electrodes [16], which hinders Li^+^ transport and ultimately limits long‑term cycling [17].

Under fast‑charging condition, the adaptability between LMA and electrolytes is exacerbated, leading to lithium dendrite growth and accelerated capacity decaying [18]. The poor safety of liquid electrolytes and the high interfacial impedance of solid‑state electrolytes greatly limit the high‑rate charging capability of lithium‑metal batteries (LMBs). Gel polymer electrolytes (GPEs) retain the low interfacial impedance similar with liquid electrolytes while offering the enhanced safety that almost bears comparison with solid‑state electrolytes [19, 20, 21, 22], making them promising for high‑rate charging LMBs. However, the application of the GPE electrolytes in fast charging LMBs is inherently limited by their low lithium‑ion transference number (*t*_Li+_) [23]. Under high‑rate charging and discharging, low *t*_Li+_ leads to pronounced concentration polarization, induces premature lithium deposition and accelerates lithium dendrite growth, and ultimately results in rapid capacity decaying [24, 25]. Therefore, developing GPE electrolytes with high *t*_Li+_ is a key strategy to fully leverage their intrinsic advantages under fast‑charging condition.

It is well recognized that *t*_Li+_ of GPE electrolytes is closely associated with the incorporated solvents in GPE electrolytes. Therefore, selecting appropriate solvents to enhance *t*_Li+_ is an effective strategy to improve fast‑charging. Among various solvents, fluoroacetonitrile (FAN) was used to enhance *t*_Li+_ by facilitating Li^+^ transport through a ligand‑channel mechanism [26, 27]. However, FAN is prone to undergo reductive polymerization on the surface of LMA, generating soluble oligomers and consequently failing to form stable interfaces. This leads to continuous consumption of FAN and formation of dead lithium, ultimately resulting in battery degradation [27‑29]. Song et al. demonstrated that the cycling performance of batteries was adversely affected when the FAN content in the electrolyte exceeded a critical ratio [27]. Liu et al. also reported that the unstable FAN‑derived solid electrolyte interface (SEI) in Li||Li cells resulted in a sharp increase in polarization voltage during lithium deposition/stripping, ultimately reaching the voltage cut‑off limit [29]. These findings indicate that the interface incompatibility between FAN and LMA hinders the realization of the intrinsic advantages offered by FAN in LMBs. To date, no effective strategy has been reported to address this issue.

This study reports an effective strategy to solve the interfacial incompatibility issues of FAN in LMBs by forming a stable SEI layer and proposes a fast‑charging gel polymer electrolyte (GPE‑FN). The proposed GPE‑FN electrolyte is composed of FAN, fluoroethylene carbonate (FEC), LiNO_3_, lithium bis(trifluoromethanesulfonyl)imide (LiTFSI), and a pentaerythritol tetraacrylate (PETEA)‑derived polymer backbone, in which FEC and LiNO_3_ are to form a stable SEI on the LMA surface to suppress the continuous consumption of FAN and facilitate interfacial Li^+^ transport, FAN is to increase *t*_Li+_ of the GPE‑FN electrolyte, and the polymer backbone is to immobilize the liquid components, respectively. Electrochemical measurements show that the GPE‑FN electrolyte achieves a high ionic conductivity (σ) of 1.66 mS cm^−1^ and a high *t*_Li+_ of 0.848 at 25°C. Both the LiFePO_4_ (LFP)||Li and LiNi_0.8_Co_0.1_Mn_0.1_O_2_ (NCM811)||Li cells based on the GPE‑FN electrolyte achieve 4100 cycles with 80.3% capacity retention and 800 cycles with 79.5% capacity retention at 5C, respectively, exhibiting excellent fast‑charging performance. Furthermore, full cells with high‑loading cathodes exhibit stable cycling, highlighting the promising potential of the GPE‑FN electrolyte. This work offers an effective strategy for designing GPE electrolytes toward fast‑charging LMBs.

## 2 Results and Discussion

**FIGURE 1:**
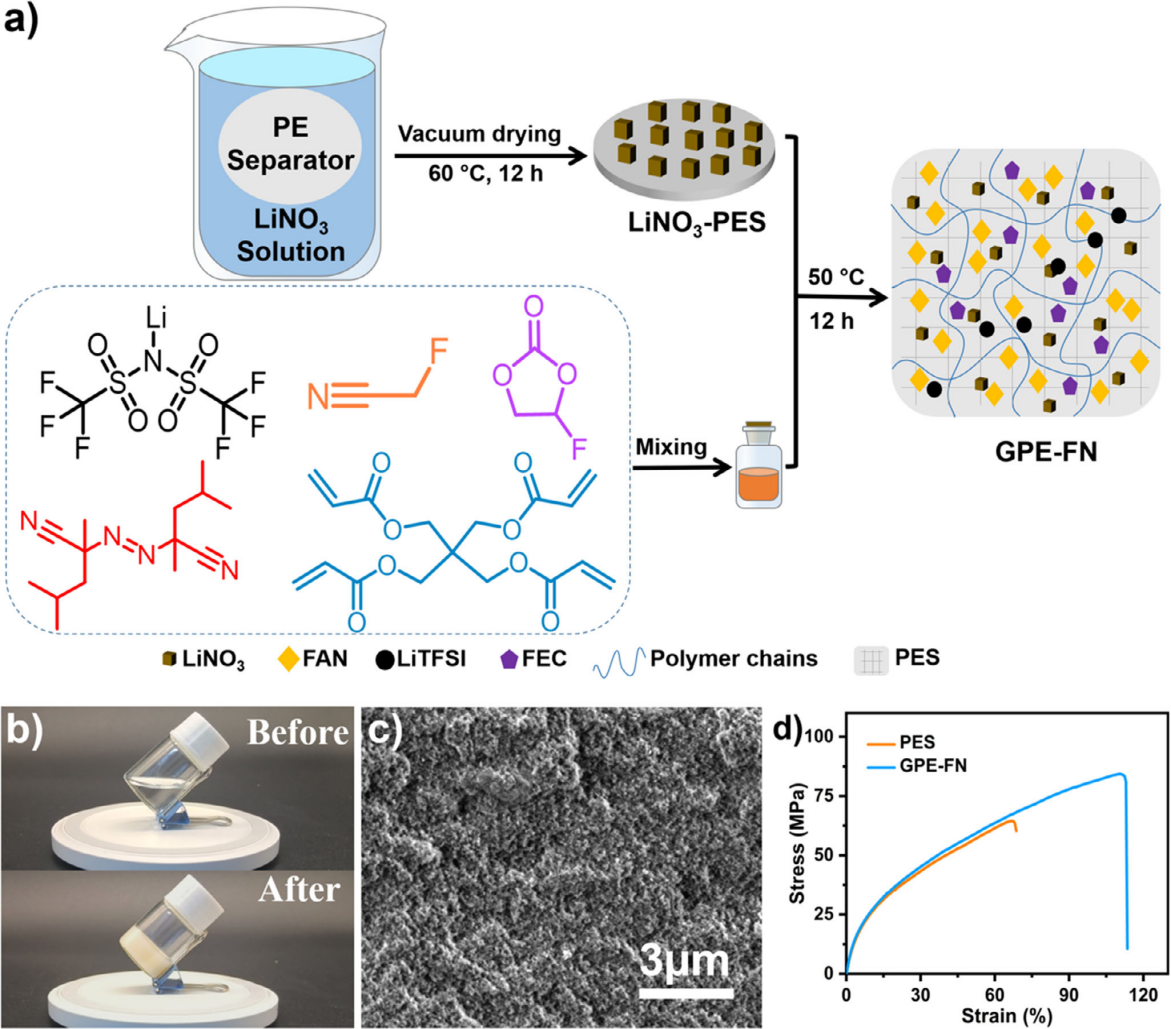
Schematic synthesis of the GPE‑FN electrolyte (a), the precursor solution before and after polymerization (b), SEM image of the GPE‑FN networks (c), tensile stress‑strain curves of the PES separator and the GPE‑FN electrolyte (d).

The synthetic procedure for the GPE‑FN electrolyte is detailed in the experimental section and illustrated in Figure 1a. First, the polyethylene separator (PES) was soaked in a LiNO_3_ solution until adsorption saturation, then taken out and dried to obtain the LiNO_3_‑modified polyethylene separator (LiNO_3_‑PES) [30, 31]. A precursor solution composed of FAN, FEC, PETEA, LiTFSI and azobis(isoheptanenitrile) (ABVN) in a specific proportion was introduced into the prepared LiNO_3_‑PES separator, and the cell was assembled. Finally, the cell was heated at 50°C to initiate the polymerization, forming GPE‑FN electrolyte and the corresponding assembled cell. For comparison, the reference electrolytes without LiNO_3_ (GPE‑F), without FEC (GPE‑N), and without both FEC and LiNO_3_ (GPE‑0) were also prepared.

The surface morphology of the PES and LiNO_3_‑PES separators was characterized using scanning electron microscope (SEM). As shown in Figure S1, the PES separator exhibits an interwoven and uniform fibrous networks with a clean fiber surface and no visible particulates. In comparison, the LiNO_3_‑PES separator shows abundant LiNO_3_ particles uniformly adhered to the fiber surface. By energy‑dispersive spectral (EDS) analysis of the PES and LiNO_3_‑PES separators, a much stronger N element signal is observed on the LiNO_3_‑PES separator compared to the unmodified PES separator. These results confirm the successful adsorption of LiNO_3_ onto the LiNO_3_‑PES separator (Figure S2). After polymerization at 50°C for 12 h, the precursor solution changes from a colorless liquid into a white solid of the crosslinked polymer networks of the GPE‑FN electrolyte (Figure 1b). The GPE‑FN network morphology was characterized using SEM, which reveals a uniform and porous structure (Figure 1c). This porous structure helps the liquid components to be homogeneously dispersed within the polymer networks, thereby ensuring uniform Li^+^ transport in the GPE‑FN electrolyte. By comparing the Fourier‑transform infrared (FTIR) spectra before and after polymerization (Figure S3), a significant decrease in intensity at 1634 cm^−1^ for the characteristic C = C absorption peak is observed [32], indicating that most of the PETEA monomers are polymerized to form the crosslinked polymer networks. The mechanical property of the GPE‑FN electrolyte was further evaluated. The tensile stress‑strain curves (Figure 1d) show that the PE separator has a tensile strength of 64.47 MPa, a fracture strain of 66.42%, and a tensile modulus of 435 MPa. In contrast, the GPE‑FN electrolyte has a tensile strength of 84.29 MPa, a fracture strain of 110.2%, and a tensile modulus increased to 492 MPa. This enhanced mechanical strength is attributed to the crosslinked gel‑like polymeric networks, which endow the GPE‑FN electrolyte with superior mechanical robustness [33].

**FIGURE 2:**
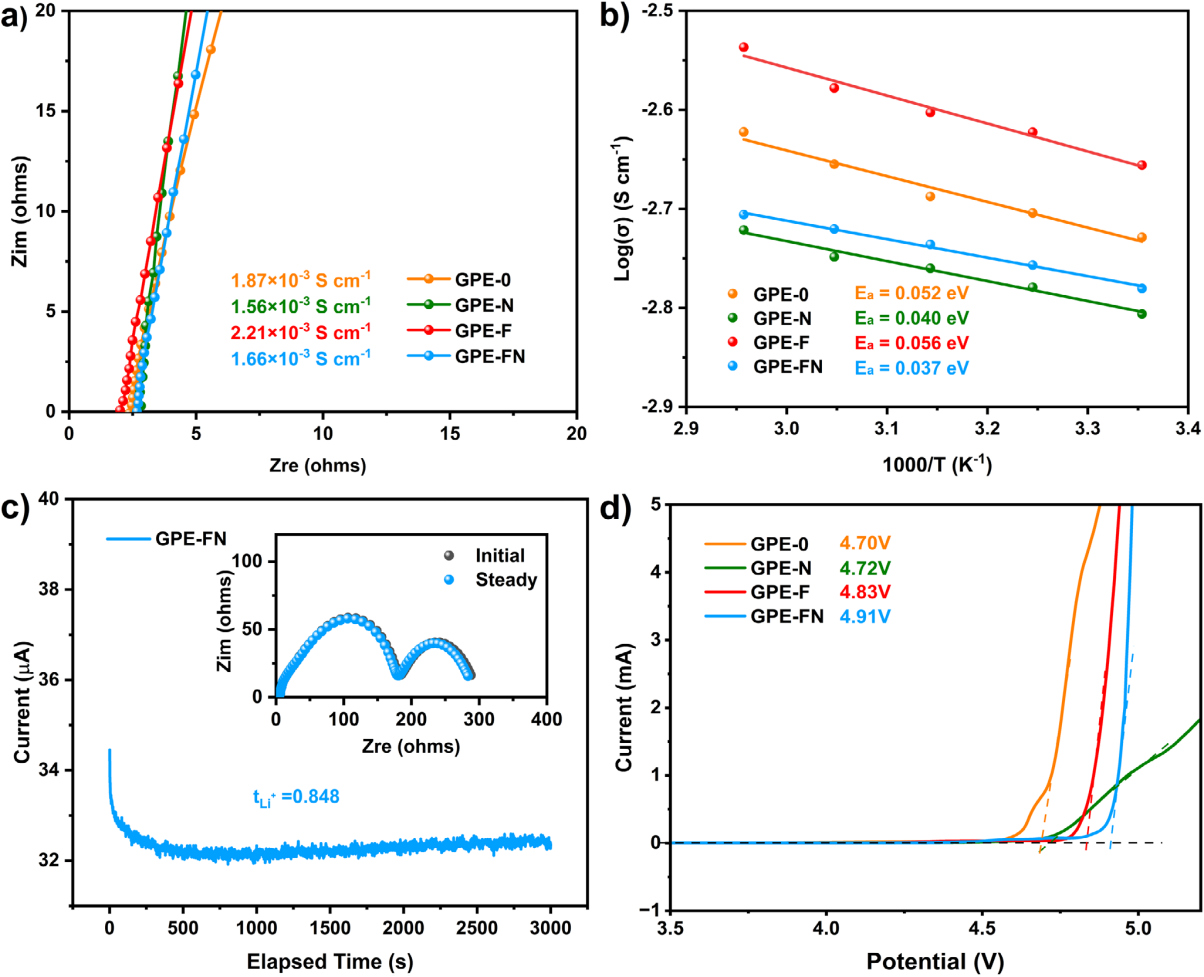
Nyquist plots of SS|GPE‑FN|SS, SS|GPE‑F|SS, SS|GPE‑N|SS, and SS|GPE‑0|SS cells at 25°C (a), σ for different GPE electrolytes at temperature ranging from 25 to 65°C (b), the chronoamperometry profile of the Li|GPE‑FN|Li cell (inset: EIS before and after polarization) (c), LSV curves of the Li|GPE‑FN|SS, Li|GPE‑F|SS, Li|GPE‑N|SS, and Li|GPE‑0|SS cells (d).

Steel (SS)||SS cells were assembled and the ionic conductivity (σ) of GPE electrolytes was measured by electrochemical impedance spectroscopy (EIS) and calculated using Equation S1. As shown in Figure 2a, σ of the GPE‑FN electrolyte at 25°C is 1.66 mS cm^−1^, which is comparable to the GPE‑0 electrolyte. All GPE electrolytes exhibit very low activation energy (*E*_a_), and the GPE‑FN electrolyte has the lowest *E*_a_ at 0.037 eV (Figure 2b), reflecting its exceptionally low Li^+^ transport resistance. *t*_Li+_ is another key parameter that characterizes the Li^+^ transport capability of GPE electrolytes. The *t*_Li+_ of the GPE‑FN electrolyte is calculated to be as high as 0.848, and *t*_Li+_ of other GPE electrolytes also exceeds 0.8 (Figure 2c; Figure S4). The high *t*_Li+_ effectively suppresses concentration polarization during fast charging, which is crucial for mitigating lithium dendrite growth and ensures fast‑charging performance.

The oxidative stability of the GPE electrolytes was evaluated by linear sweep voltammetry (LSV). The oxidation potential of GPE‑FN, GPE‑F, and GPE‑N electrolytes is 4.91, 4.83, and 4.72 V, respectively, all higher than that of the GPE‑0 electrolyte (4.70 V) (Figure 2d), indicating that FEC and LiNO_3_ enhance the oxidative stability of the GPE electrolytes. Electrochemical floating analysis (EFA) was further conducted to evaluate the oxidative stability of the GPE electrolytes under practical charging condition. The GPE‑FN electrolyte remains stable below 4.40 V. Given that most LMBs typically operate below 4.30 V, this stability window is sufficient for practical application (Figure S5).

**FIGURE 3:**
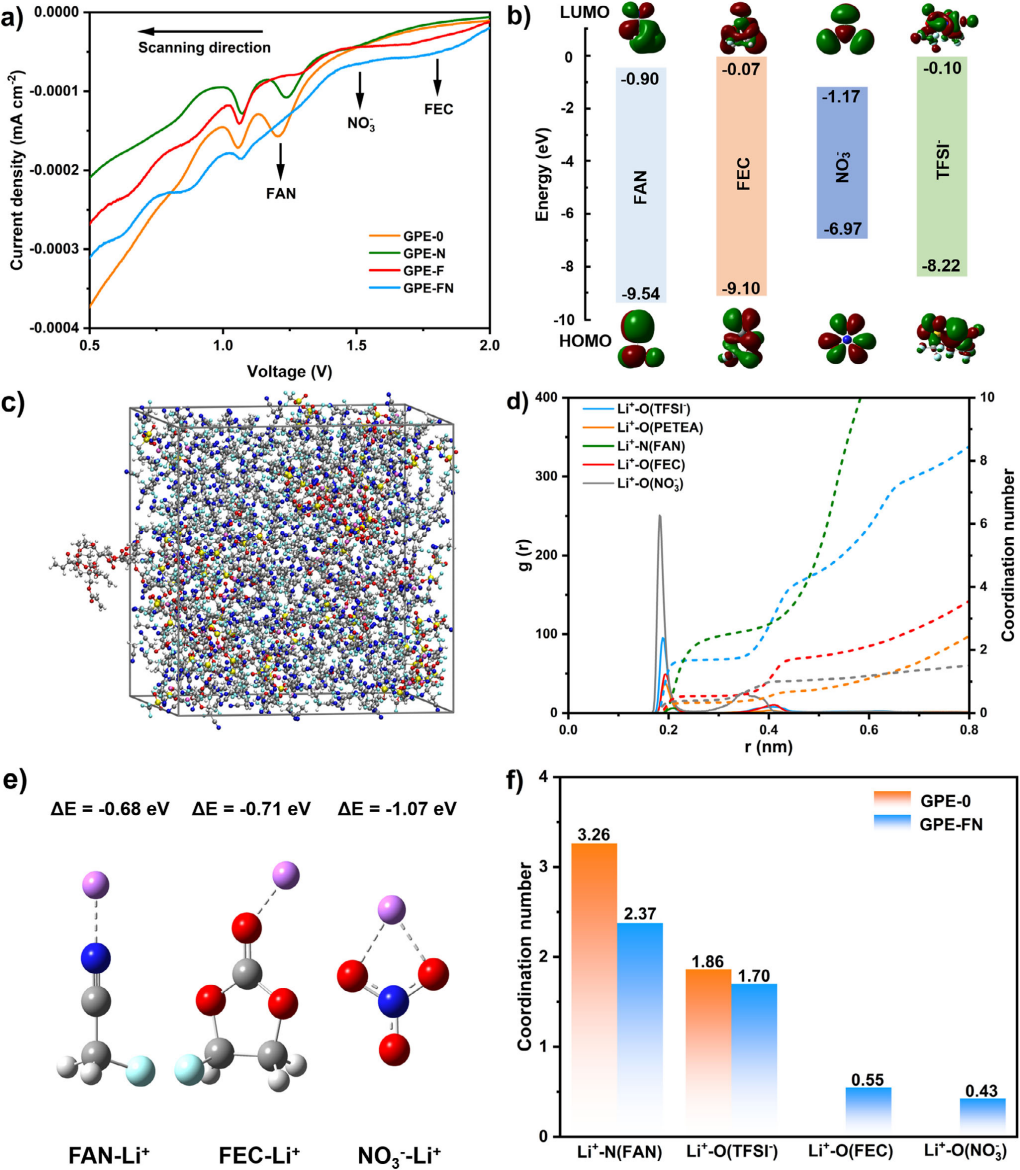
CV curves of Li|GPE‑FN|Cu, Li|GPE‑F|Cu, Li|GPE‑N|Cu, and Li|GPE‑0|Cu cells (a), LUMO and HOMO of FAN, FEC, NO_3_^−^, and TFSI^−^ (b), GPE‑FN snapshot obtained by MD simulation (c), RDF and CN of Li^+^ in the GPE‑FN electrolyte (d), computational binding energies of FAN, NO_3_^−^, and FEC with Li^+^ (e), and the CN of Li^+^ with solvent molecules and anions in GPE‑FN and GPE‑0 electrolytes (f).

FEC and NO_3_^−^ must be reduced to form a stable SEI on LMA prior to FAN undergoing reduction, thereby effectively preventing the continuous consumption of FAN. To investigate the practical reduction of GPE electrolytes, Li||Cu cells were assembled employing the GPE‑FN electrolyte as well as the reference GPE‑F, GPE‑N, and GPE‑0 electrolytes, and the cyclic voltammetry (CV) was checked (Figure 3a). The CV curve of the GPE‑0 electrolyte displays a reduction peak at 1.2 V, corresponding to the reduction of FAN. In comparison, the CV curve of the GPE‑F electrolyte shows two reduction peaks at 1.8 V corresponding to FEC and at 1.3 V corresponding to FAN [34], and the intensity of the FAN‑related reduction peak is significantly reduced. This suggests that the preferential reduction of FEC effectively prevents the reduction of FAN. For the CV curve of the GPE‑FN electrolyte, which contains both LiNO_3_ and FEC, the reduction peaks at 1.8 and 1.5 V for FEC and NO_3_^−^ are observed [35], and the FAN‑related peak is nearly absent. These results demonstrated that LiNO_3_ and FEC work collectively to prevent the reduction of FAN.

Based on the density functional theory (DFT), the lowest unoccupied molecular orbital (LUMO) of FAN, FEC, and NO_3_^−^ is −0.90, −0.07, and −1.17 eV, respectively (Figure 3b). This shows a discrepancy with the actual reduction sequence observed in the GPE‑FN electrolyte, indicating that the practical reduction is determined not solely by thermodynamics. Molecular dynamics (MD) simulation of the GPE‑FN and GPE‑0 electrolytes is further conducted to investigate the reduction of FAN, FEC, and NO_3_^−^. Snapshots of the MD simulation for the GPE‑0 and GPE‑FN electrolytes are shown in Figure 3c and Figure S6. The calculated radial distribution function (RDF) and coordination number (CN) are displayed in Figure 3d and Figure S7, assuming that Li^+^ is coordinated by anions and solvent molecules, both of which form a solvation shell. In the solvation shell, the coordination distances between Li^+^ and the O atoms of FEC and NO_3_^−^ are 0.194 and 0.188 nm, respectively, both shorter than the Li^+^‑N distance of 0.210 nm for FAN. These results indicate that FEC and NO_3_^−^ preferentially occupy the inner region of the solvation shell compared with FAN, suggesting stronger coordination with Li^+^ [36]. This solvation structure is consistent with the binding energy calculation (Figure 3e) and it implies a lower Li^+^ desolvation energy barrier for FEC and NO_3_^−^ during the interfacial reaction compared with FAN, thereby favoring the reduction of FEC and NO_3_^−^ over FAN [37]. In the GPE‑0 electrolyte, the CNs of FAN and TFSI^−^ are 3.26 and 1.86, respectively. In GPE‑FN, the CNs decrease to 2.37 and 1.70, while the CNs of FEC and NO_3_^−^ further increase to 0.55 and 0.43, respectively (Figure 3f). This indicates that through competitive coordination with Li^+^, FEC and NO_3_^−^ displace FAN from the solvation shell and alter the solvation structure of Li^+^ [38]. This means that less FAN will be delivered to the LMA surface, thereby lowering its reduction, and therefore FEC and NO_3_^−^ will be transported to the LMA surface and preferentially reduced there, preventing the reduction of FAN at the interface.

**FIGURE 4:**
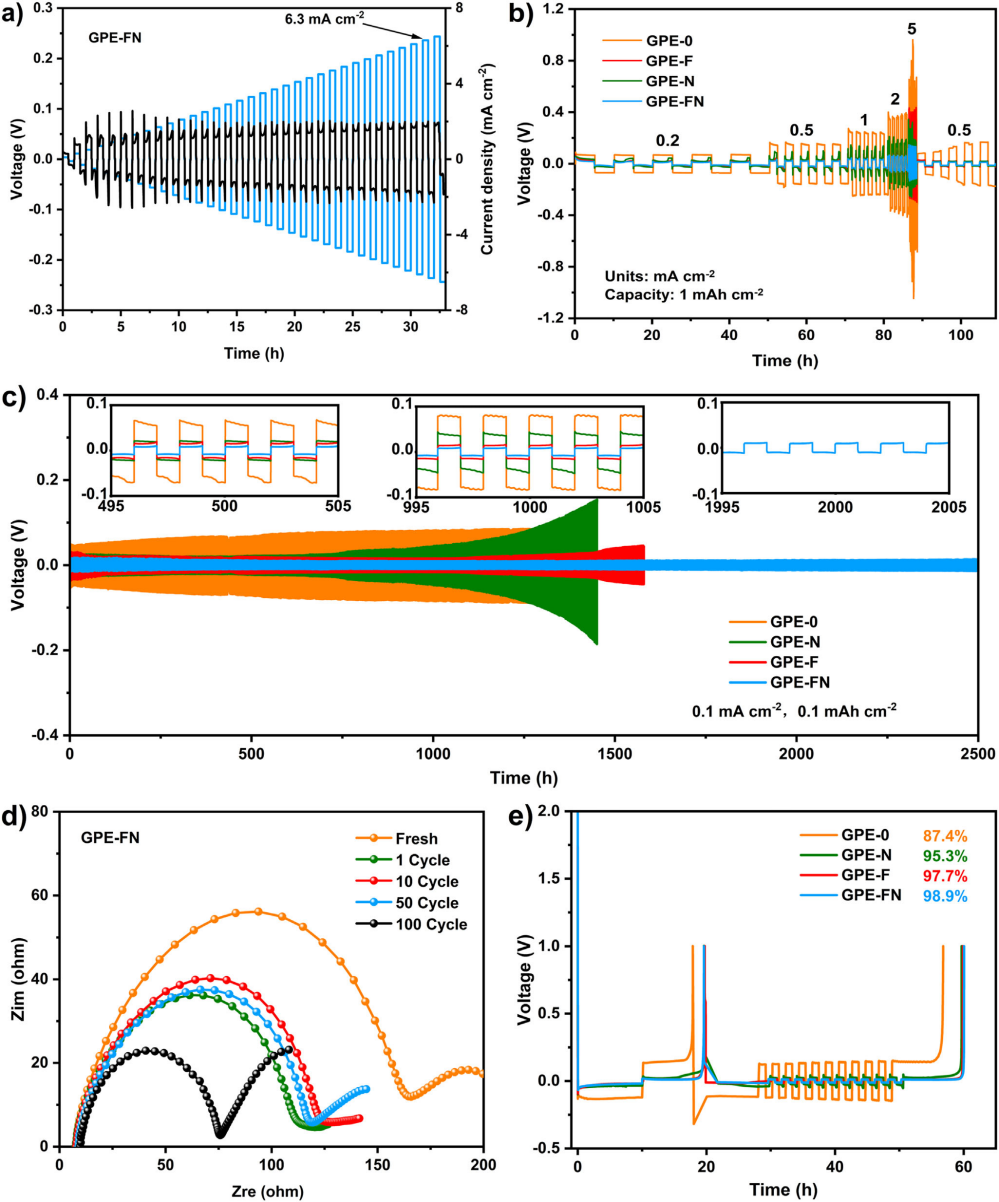
CCD plots of the Li|GPE‑FN|Li cell under step‑up current densities with a constant Li plating and stripping time of 0.5 h (a), rate capability of Li|GPE‑FN|Li, Li|GPE‑F|Li, Li|GPE‑N|Li, and Li|GPE‑0|Li cells at 1 mAh cm^−2^ (b), long‑time cycling voltage profiles of the Li|GPE‑FN|Li, Li|GPE‑F|Li, Li|GPE‑N|Li, and Li|GPE‑0|Li cells at 0.1 mA cm^−2^ and 0.1 mAh cm^−2^ (inset: corresponding enlarged view of voltage curves at different cycling stages) (c), EIS spectra of the Li|GPE‑FN|Li cell before and after various cycle numbers (d), CE measurement of the Li|GPE‑FN|Cu, Li|GPE‑F|Cu, Li|GPE‑N|Cu, and Li|GPE‑0|Cu cells (e).

To evaluate the interfacial stability of the GPE electrolytes during lithium deposition/stripping, Li||Li cells were assembled. The Li||Li cells employing GPE‑FN, GPE‑F, GPE‑N, and GPE‑0 electrolytes achieve a critical current density (CCD) of 6.9, 3.3, 4.5, and 2.9 mA cm^−2^, respectively (Figure 4a; Figure S8). The highest CCD of the Li||Li cell employing the GPE‑FN electrolyte indicates excellent resistance to lithium dendrite growth and robust interfacial stability. The Li||Li cell employing the GPE‑FN electrolyte also exhibits excellent rate capability, with the overpotential increasing from 9 mV to 98 mV as the current density increasing from 0.2 to 5 mA cm^−2^, representing the lowest value among all GPE electrolytes, while the Li||Li cell employing the GPE‑0 electrolyte quickly suffers micro‑short circuit during charging and discharging at a current density of 5 mA cm^−2^ (Figure 4b). This indicates that it achieves fast and stable Li^+^ transport at the interface in the Li||Li cell employing the GPE‑FN electrolyte. In long‑term cycling test, the overpotential of the Li|GPE‑FN|Li, Li|GPE‑F|Li, and Li|GPE‑N|Li cells shows a decreasing trend during the initial cycles at a current density of 0.1 mA cm^−2^ and an areal capacity of 0.1 mAh cm^−2^. The Li|GPE‑FN|Li cell exhibits the lowest overpotential, with an initial overpotential of only 17 mV, and the overpotential does not significantly increase during 2500 h cycling, demonstrating good interfacial stability. In contrast, the overpotential of the Li|GPE‑0|Li cell gradually increases, exceeding 80 mV after 910 h cycling (Figure 4c). These results can be attributed to the SEI layer formed by FEC and NO_3_^−^, which effectively insulates the reduction of FAN and lowers the interfacial transport resistance of Li^+^.

EIS was conducted on Li||Li cells after different plating/stripping cycles (Figure 4d; Figure S9). Figure S10 shows the equivalent circuit diagram corresponding to the impedance fitting of the Li||Li cells. The interface resistance of the Li|GPE‑0|Li cell increases from 184.7 to 186.1 Ω after 100 cycles, which is ascribed to by‑products generated from FAN reduction. In contrast, the interfacial impedance of the Li|GPE‑FN|Li, Li|GPE‑F|Li, and Li|GPE‑N|Li cells decreases after 100 cycles, indicating that the SEI layer generated by the reduction of FEC and NO_3_^−^ effectively prevents the generation of harmful by‑products. Tafel curves show that the Li|GPE‑FN|Li cell has an exchange current density of 1.153 mA cm^−2^ much higher than Li|GPE‑F|Li (0.241 mA cm^−2^), Li|GPE‑N|Li (0.328 mA cm^−2^) and Li|GPE‑0|Li (0.194 mA cm^−2^), further confirming that the GPE‑FN electrolyte exhibits faster Li^+^ reaction kinetics and superior interfacial compatibility (Figure S11) [39, 40]. According to the Aurbach method [41], Li||Cu cells were assembled to test the coulombic efficiency (CE) (Figure 4e; Equation S4). The CE of Li|GPE‑FN|Cu, Li|GPE‑F|Cu, Li|GPE‑N|Cu and Li|GPE‑0|Cu cells is calculated to be 98.9%, 97.7%, 95.3% and 87.4%, respectively. The highest CE of the Li|GPE‑FN|Cu cell is attributed to the SEI layer formed by FEC and NO_3_^−^, which effectively prevents the FAN reduction and formation of dead lithium. Furthermore, during the Li||Cu cell cycling, the average CE of the Li|GPE‑0|Cu cell drops to 79.9% after just 62 cycles, whereas the Li|GPE‑FN|Cu cell maintains a 97.9% CE throughout the cycling, demonstrating significantly superior cycling reversibility (Figure S12) [42].

**FIGURE 5:**
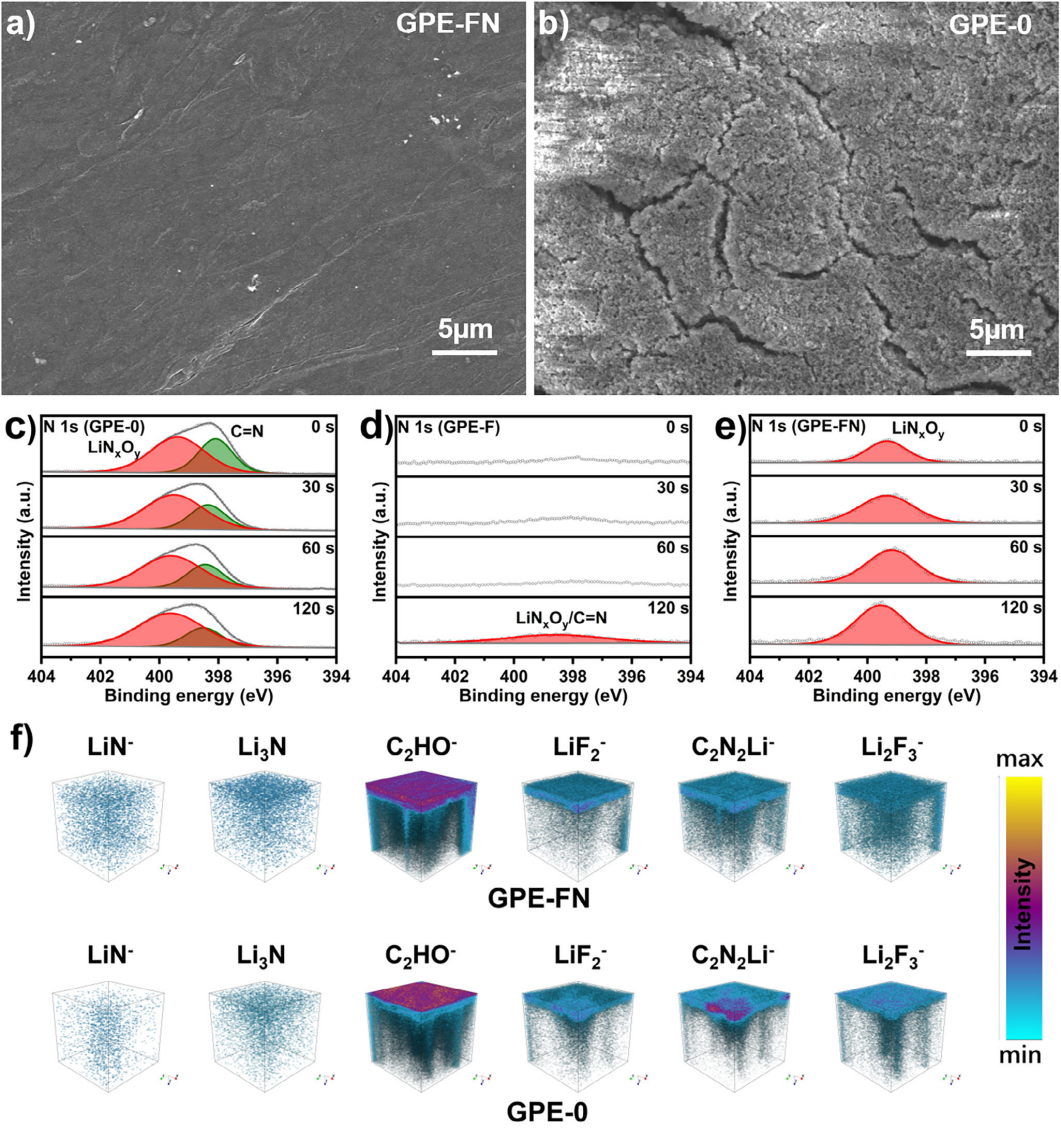
Surface SEM images for lithium metal electrodes of the Li|GPE‑FN|Li (a) and Li|GPE‑0|Li (b) cells after 100 cycles at 0.1 mA cm^−2^ and 0.1 mAh cm^−2^, XPS depth profiles of N 1s spectra for the cycled lithium metal electrodes employing the electrolytes of GPE‑0 (c), GPE‑F (d), and GPE‑FN (e), TOF‑SIMS 3D mapping images of several representative secondary ion fragments for the cycled lithium metal electrodes employing GPE‑FN and GPE‑0 electrolytes (f).

The surface morphology of lithium metal electrodes in Li||Li cells after cycling was examined by SEM. As shown in Figure 5a, the surface of the lithium metal electrodes in the cycled Li|GPE‑FN|Li cells is uniform and dense, without obvious dendrites. In contrast, a few dendrites in the Li|GPE‑F|Li cell (Figure S13) and a cracked SEI layer in the cycled Li|GPE‑0|Li cell are observed (Figure 5b). These confirm that the coordination of FEC and LiNO_3_ reduces the interfacial by‑products and dendrite formation in the Li|GPE‑FN|Li cells.

XPS was used to characterize the SEI layer on lithium metal electrodes, and the atom percentages of C, N, O, and F elements were analyzed. For the cycled Li|GPE‑0|Li cells, a characteristic peak at 398.4 eV attributed to the N atom in the C = N component is observed in the SEI layer (Figure 5c; Figure S14c) [43]. This C = N component originates from the oligomers and trimers [44], which are formed by the reduction of FAN. With the sputtering time increasing, the N atom percentage gradually decreases, indicating the enhanced reduction of FAN during SEI formation (Figure S15a). For the cycled Li|GPE‑F|Li cells, the SEI layer exhibits a higher C atom percentage but a much lower N atom percentage at any given sputtering time, reflecting a higher organic content in the SEI layer (Figure 5d; Figure S15a,c). This organic content is primarily composed of polycarbonate (C = O) and polyether (C‐O) components derived from FEC decomposition (Figure S16d) [45]. For example, the N atom with a characteristic signal at 398.6 eV at 120 s sputtering time is as low as 0.63% atom percentage, while no significant signal of the N atom is observed at 60 s (Figure 5d; Figure S14b). These results indicate that FEC is preferentially reduced to form an organic‑rich SEI layer in the Li|GPE‑F|Li cells, which effectively prevents the reduction of FAN [45]. In comparison, for the cycled Li|GPE‑FN|Li cells, the SEI layer shows a significant increase in the atom percentages of N and O, alongside a decrease in C (Figure S15c,d). Besides, the reappearance of the N atom signal after 60 s sputtering time (Figure S14a) and the characteristic peaks for the N atom in LiN_x_O_y_ (399.2 eV) and the O atom in Li_2_O (528.9 eV), confirms the NO_3_^−^ reduction in the SEI and construction of an inorganic‑rich SEI layer (Figure 5e; Figure S16). Three‑dimensional depth profiling of the SEI layer was further performed using TOF‑SIMS (Figure 5e; Figures S17 and S18). For the Li|GPE‑0|Li cells, the SEI layer exhibits a strong and unevenly distributed signal for the C_2_N_2_Li^−^ fragments originating from the reduction of FAN. In contrast, the SEI layer of the Li|GPE‑FN|Li cell shows an extremely weak C_2_N_2_Li^−^ signal but a stronger, more uniform and deeper longitudinal distribution of the C_2_HO^−^ fragments [36]. This demonstrates that the reduction products of FEC preferentially cover the lithium metal electrode surface, thereby inhibiting further reduction of FAN. Additionally, the signals of ionic fragments such as Li_3_N and LiF_2_^−^ are significantly enhanced, indicating a higher content of the inorganic components such as Li_3_N and LiF, which possess high ionic conductivity and mechanical strength, thereby improving Li^+^ transport and interfacial stability [46, 47].

**FIGURE 6:**
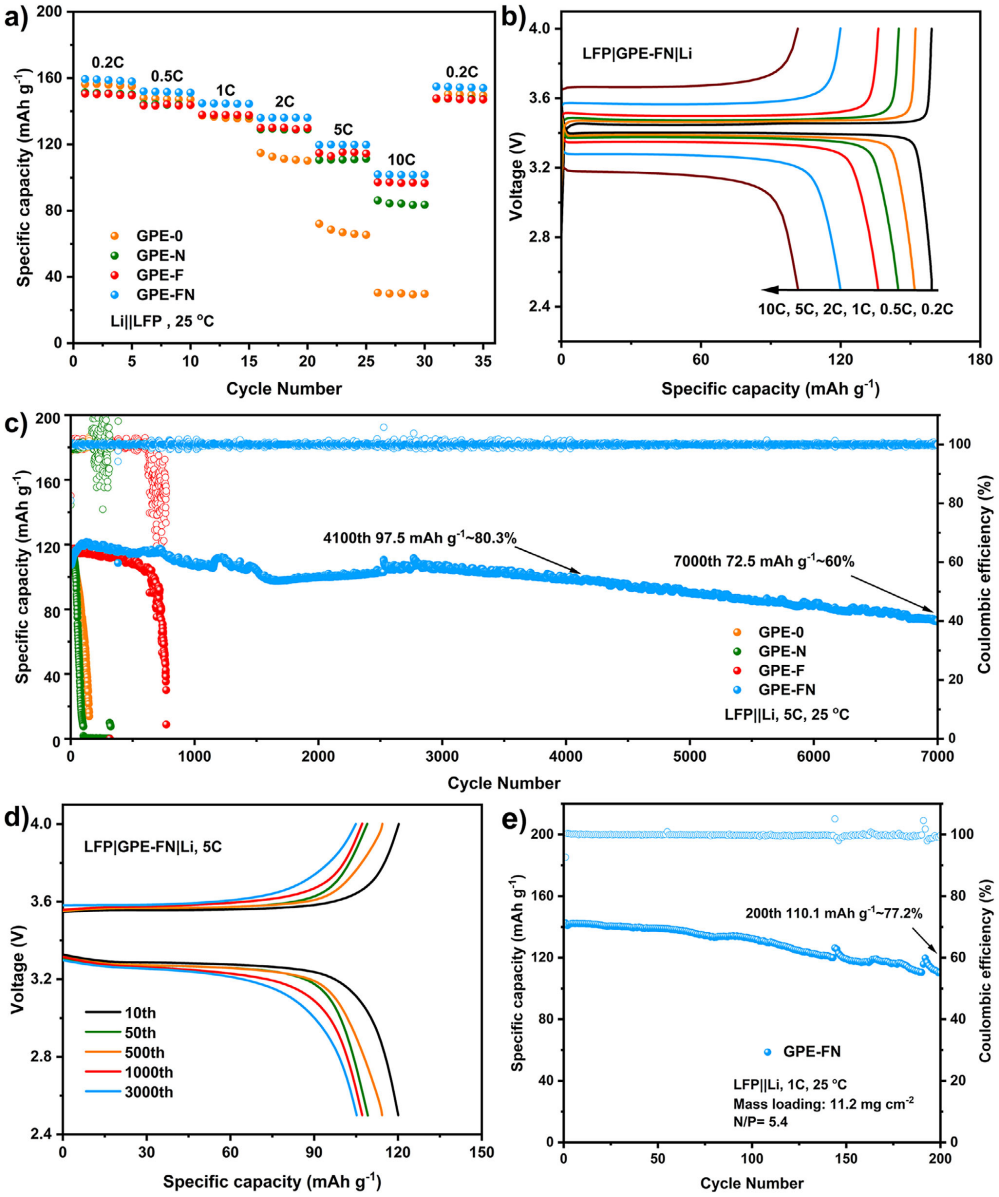
Rate capabilities of the LFP|GPE‑FN|Li, LFP|GPE‑F|Li, LFP|GPE‑N|Li, and LFP|GPE‑0|Li cells at 25°C (a), the charging/discharging curves from 0.2C to 10C of the LFP|GPE‑FN|Li cell (b), long‑term cycling of the LFP|GPE‑FN|Li, LFP|GPE‑F|Li, LFP|GPE‑N|Li, and LFP|GPE‑0|Li cells at 5C and 25°C (c), the charging/discharging curves of the LFP|GPE‑FN|Li cell at 5C (d), long‑term cycling of the LFP|GPE‑FN|Li full cell at 1C and 25°C (e).

The LFP||Li cells were assembled and their rate capability and cycling performance were tested. The LFP|GPE‑FN|Li cell demonstrates excellent rate capability and its average discharging capacity decreases from 158.7 to 101.7 mAh g^−1^ with the rate increasing from 0.2C to 10C, much better than the cells employing other reference GPE electrolytes of GPE‑F, GPE‑N, and GPE‑0 (Figure 6a,b). In the long‑term cycling at 2C, the LFP|GPE‑FN|Li cell demonstrates good cycling stability, showing 81.4% capacity retention after 3000 cycles and a maximum discharging specific capacity of 130.3 mAh g^−1^ (Figure S19). Furthermore, in the case of fast‑charging at 5C, as shown in Figure 6c,d, the LFP|GPE‑FN|Li cell shows a maximum discharging specific capacity of 121.6 mAh g^−1^ with 80.3% capacity retention after 4100 cycles, along with a low polarization and flat voltage plateau, and maintains a CE essentially above 99.0%. In contrast, the cells employing reference GPE‑F, GPE‑N, and GPE‑0 electrolytes suffer rapid capacity fade, falling below 80% capacity retention after only 657, 48, and 85 cycles, respectively. The LFP|GPE‑0|Li cell, particularly undergoes complete capacity failure after 178 cycles due to continuous consumption of FAN.

To assess the GPE‑FN electrolyte in practical application, the full cell with a low N/P ratio of 5.4 was further assembled using a high‑loading LFP cathode (11.2 mg cm^−2^) and thin LMA (50 µm), and its charging/discharging at 1C was tested. The full cell achieves an initial discharging specific capacity of 142.6 mAh g^−1^ with a CE of 92.61%, and retains a discharging specific capacity of 110.1 mAh g^−1^ after 200 cycles with a 77.2% capacity retention, while showing a flat voltage plateau during the first 50 cycles (Figure 6e; Figure S20), demonstrating good potential for practical application.

**FIGURE 7:**
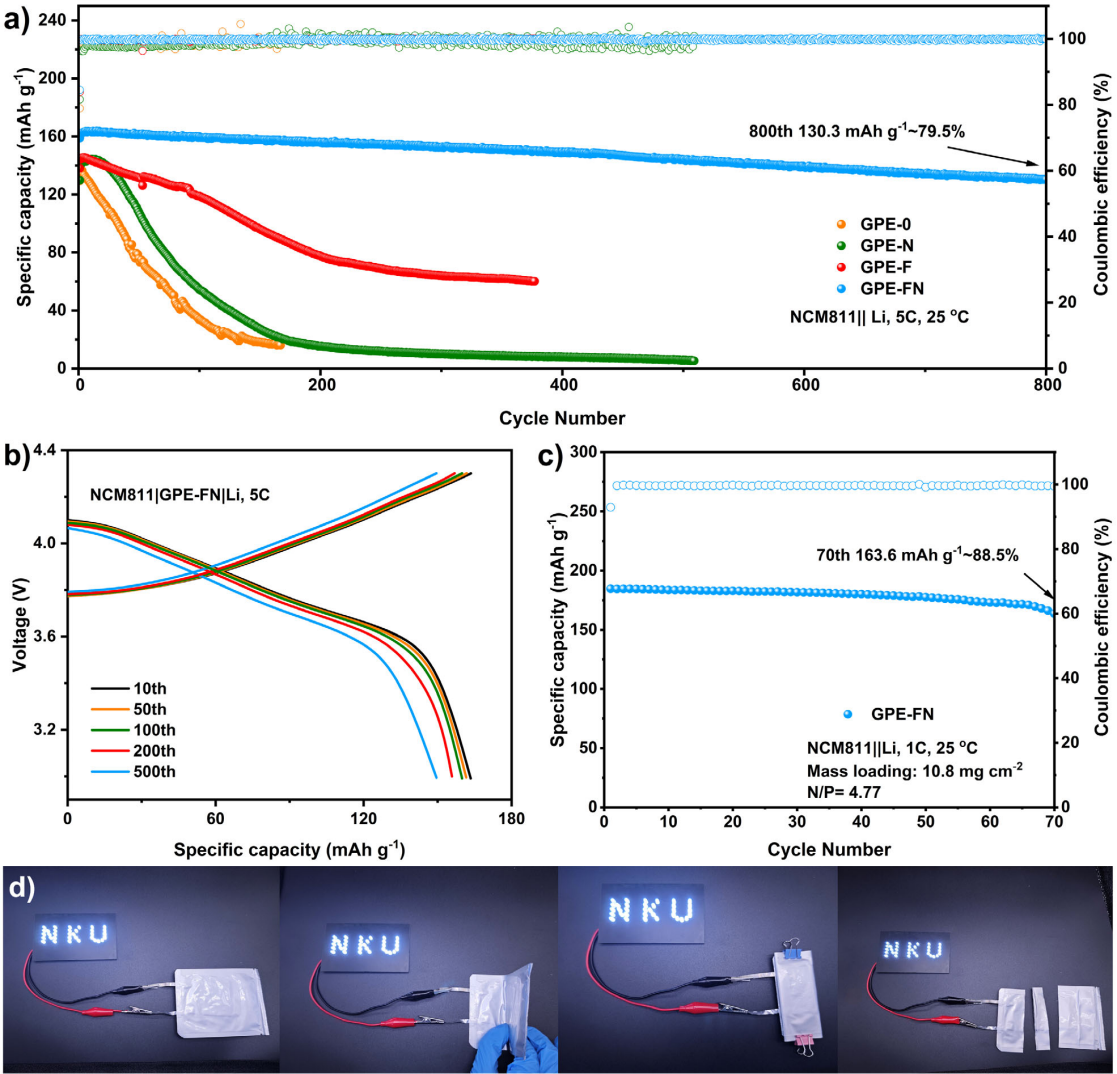
Long‑term cycling of the NCM811|GPE‑FN|Li, NCM811|GPE‑F|Li, NCM811|GPE‑N|Li, and NCM811|GPE‑0|Li cells at 5C and 25°C (a), the charging/discharging curves of the NCM811|GPE‑FN|Li cell at 5C (b), long‑term cycling of the NCM811|GPE‑FN|Li full cell at 1C and 25°C (c), the assembled LFP|GPE‑FN|Li pouch cell illuminates the LED light under flatting, bending 90 ^o^, folding, and cutting conditions (d).

The GPE‑FN electrolyte was further evaluated for the cell employing the high‑voltage cathode of LiNi_0.8_Co_0.1_Mn_0.1_O_2_ (NCM811). As shown in Figure 7a,b, the NCM811|GPE‑FN|Li cell exhibits a maximum discharging specific capacity of 163.8 mAh g^−1^ and a 79.5% capacity retention after 800 cycles at 5C (1C = 200 mAh g^−1^), with a slow increase in polarization voltage during cycling and a CE remaining essentially above 99.5%. Additionally, the NCM811|GPE‑FN|Li cell achieves stable cycling over 1000 cycles at 2C (Figure S21). In contrast, the reference NCM811|GPE‑0|Li cell drops below 80% capacity retention after only 34 cycles at 5C, and its CE exhibits significant fluctuations. In addition, the full cell with a low N/P ratio of 4.77 was assembled using the GPE‑FN electrolyte, high‑loading NCM811 cathode (11.2 mg cm^−2^) and thin LMA (50 µm). As shown in Figure 7c and Figure S22, the full cell keeps 88.5% capacity retention after 70 cycles at 1C with a CE close to 100% and its polarization voltage increases slowly during the first 50 cycles. These results indicate that the GPE‑FN electrolyte is compatible with the NCM811 high‑voltage cathode. Moreover, a comparative evaluation with recently reported GPE electrolytes demonstrates the favorable performance of the GPE‑FN electrolyte (Table S1).

Finally, the pouch cells were assembled to evaluate the reliability and safety of the GPE‑FN electrolyte. The LFP|GPE‑FN|Li and NCM811|GPE‑FN|Li cells exhibit stable performance for 20 and 25 cycles, respectively, with no occurrence of short circuits or other failures (Figure S23). Furthermore, the LFP|GPE‑FN|Li pouch cell can successfully power an LED light even under harsh mechanical conditions, including being bent at 90 °, folded, and cut (Figure 7d). These results confirm the excellent safety and reliability of the GPE‑FN electrolyte employed in the pouch cells.

## 3 Conclusion

In summary, a gel polymer electrolyte (GPE‑FN) containing FAN, FEC, and LiNO_3_ was prepared for fast‑charging LMBs. In the GPE‑FN electrolyte, the combined use of FEC and LiNO_3_ promotes the formation of a stable SEI layer on the LMAs, which effectively prevents the continuous consumption of FAN and facilitates superior Li^+^ transport across the interface. The GPE‑FN electrolyte exhibits a high σ of 1.66 mS cm^−1^ and a high *t*_Li+_ of 0.848 at 25°C. The Li|GPE‑FN|Li cell exhibits a CCD of 6.9 mA cm^−2^ and achieves over 2500 h of deposition/stripping cycling at a current density of 0.1 mA cm^−2^. Notably, the LFP|GPE‑FN|Li cell runs 4100 cycles at 5C, with a maximum discharging specific capacity of 121.6 mAh g^−1^. In addition, the GPE‑FN electrolyte demonstrates good compatibility with the high‑voltage NCM811 cathode. The NCM811|GPE‑FN|Li cell retains 79.5% capacity after 800 cycles at 5C and exhibits a highest discharging specific capacity of 163.8 mAh g^−1^. Furthermore, full cells based on GPE‑FN electrolyte with high‑loading LFP and NCM811 cathodes exhibit stable cycling at 1C, demonstrating the practical application of the GPE‑FN electrolyte. Overall, this work presents a promising GPE for LMBs with long‑term cycling stability and excellent fast‑charging capability, demonstrating its strong potential for practical LMB application.

## Conflicts of Interest

The authors declare no conflicts of interest.

## Supporting information




**Supporting File**: advs75528‐sup‐0001‐SuppMat.pdf.

## Data Availability

The data that support the findings of this study are available from the corresponding author upon reasonable request.
