# Supplementary material for: Fluoroacetonitrile‑Based Gel Polymer Electrolytes for Fast‑Charging Lithium Metal Batteries
Source: Adv Sci (Weinh). 2026 May 6;13(42):e75528. doi: 10.1002/advs.75528 (PMC13335625; doi:10.1002/advs.75528)
Supplement: Supplementary file 1 — Supporting File: advs75528‐sup‐0001‐SuppMat.pdf. [file ADVS-13-e75528-s001.pdf]

Supporting Information for:

Fluoroacetonitrile-based Gel Polymer Electrolytes for Fast-charging Lithium Metal Batteries

Zuoxin Yang,[a] Jinyuan Wang,[a] Zhe Tian,[a] Boyi Song,*[a] Fangyi Cheng,*[b,c] Wangqing Zhang*[a,b,d]

[a] Z. Yang, J. Wang, Z. Tian, B. Song,* W. Zhang*
Nankai University
Institute of Polymer Chemistry, College of Chemistry
Key Laboratory of Functional Polymer Materials of the Ministry of Education, Tianjin 300071, China.
E-mail: bysong@nankai.edu.cn, wqzhang@nankai.edu.cn

[b] F. Cheng,* W. Zhang*
Nankai University
College of Chemistry
Frontiers Science Center for New Organic Matter, Tianjin 300071, China.
E-mail: fycheng@nankai.edu.cn, wqzhang@nankai.edu.cn

[c] F. Cheng*
Nankai University
College of Chemistry
Key Laboratory of Advanced Energy Materials Chemistry of the Ministry of Education, Tianjin 300071, China.
E-mail: fycheng@nankai.edu.cn

[d] W. Zhang*
Nankai University
Tianjin Key Laboratory of Functional Polymer Materials, Tianjin 300071, China.
E-mail: wqzhang@nankai.edu.cn

Table of Contents

1. Materials……………...………………3

2. Preparation of cathodes...3

3. Preparation of electrolytes and fabrication of cells..3

4. Materials characterization..3

5. Electrochemical measurements..4

6. Computational methods..5

7. Supplementary Figures and Tables..7

8. Supplementary references..21

Materials

Fluoroacetonitrile (FAN, Anhui Senrise Technologies Co., Ltd., China), fluoroethylene carbonate (FEC, Bide Pharmatech Ltd., China), $LiNO_3$ (Shanghai Meryer Biochemical Technology Co., Ltd.), pentaerythritol tetraacrylate (PETEA, Shanghai Meryer Biochemical Technology Co., Ltd., China), lithium bis(trifluoromethanesulfonyl)imide (LiTFSI, Bide Pharmatech Ltd., China), methanol (Tianjin Bohua Reagent Co., Ltd., China), polyethylene separator (PES, 25 μm, Asahi Kasei Co.), 2,2'-azobisisoheptonitrile (Tianjin Chemical Company, China), *N*-methyl-2-pyrrolidone (NMP, Tianjin Bohua Reagent Co., Ltd., China), $LiFePO_4$ (LFP, Shenzhen Bonapu Automation Technology Co., Ltd., China), $LiNi_{0.8}Co_{0.1}Mn_{0.1}O_2$ (NCM811, Dongguan Kelu De experimental equipment Technology Co., Ltd., China), super P. carbon (Shenzhen Kejing Star Technology Co., Ltd., China), aluminum foil current collector (Shenzhen Kejing Star Technology Co., Ltd., China), poly(vinylidene difluoride) (PVDF, Kynar HSV 900, Akema), lithium metal anode (200 μm and 50 μm, China Energy Lithium Co., Ltd., China), high-loading LFP cathode (around 11.2 mg cm^{-2}, Dongguan KeluDe experimental equipment Technology Co., Ltd., China), high-loading NCM811 cathode (around 10.8 mg cm^{-2}, Dongguan Kelu De experimental equipment Technology Co., Ltd., China).

Preparation of cathodes

The cathode slurry was prepared by mixing active material (LFP or NCM811), super P. carbon, and the PVDF binder (5.0 wt% in NMP) with the mass ratio of active material: PVDF: Super P. at 8:1:1, where NMP was used as solvent. The slurry is then applied onto the surface of the aluminum foil using a scraper and dried in a convection oven at 60 °C for 3 h, followed by vacuum drying at 120 °C for 12 h to obtain the cathode. The active material loading was approximately 2 mg cm^{-2}.

The high-loading LFP and high-loading NCM811 cathodes were vacuum-dried at 120 °C for 12 h before use.

Preparation of electrolytes and fabrication of cells

The PES separator was punched into disks with a diameter of 16 mm and soaked in a methanol solution of $LiNO_3$ with a concentration of 0.20 g mL^{-1} for 12 h. Then, the PES separator was removed and vacuum-dried at 60 °C for 12 h to $LiNO_3$-modified polyethylene separator ($LiNO_3$-PES). The $LiNO_3$ loading on the $LiNO_3$-PES separator was 0.11 mg cm^{-2}. Then, a precursor solution composed of FAN, FEC, PETEA, LiTFSI, and azobis(isoheptanenitrile) (ABVN) in a specific proportion 1.40 g FAN, 0.10 g FEC, 0.10 g PETEA, 0.40 g LiTFSI, and 2.0 mg ABVN, followed by stirring until homogeneous. A total of 60 μL of the precursor solution was introduced between two layers of the PES separator, and the coin cell (CR2032) was used for battery fabrication, which was subsequently heated at 50 °C for 12 h to form the GPE-FN electrolyte. For comparison, electrolytes without $LiNO_3$ (GPE-F), without FEC (GPE-N), and without both FEC and $LiNO_3$ (GPE-0) were also prepared.

Materials characterization

Tensile stress-strain curves were obtained by performing tensile tests on a universal testing instrument (SongDun, WDW-5, China). The tensile rate employed was 10 mm min^{-1}.

Fourier transform infrared (FTIR) measurements were conducted using the TENSOR II infrared spectrometer. The spectral range was 400-4000 cm^{-1}, with a collection resolution of 2 cm^{-1}.

Scanning electron microscope (SEM) micrographs and energy-dispersive spectral (EDS) maps were acquired using a JSM-7500F field-emission SEM and a Phenom Pure instrument, respectively, both operated at an accelerating voltage of 5 kV.

X-ray photoelectron spectroscopy (XPS) analysis was performed using an Axis Ultra DLD spectrometer (Kratos Analytical Ltd., UK).

Time-of-flight secondary ion mass spectrometer (TOF-SIMS) measurements were performed using a TOF.SIMS 5-100 instrument (IONTOF GmbH). A pulsed ion beam of Bi^{3+} at 30 keV in high-current mode was used for in-depth analysis, and the samples were sputtered with a Cs^+ beam at 1 keV over an area of 200 μm × 200 μm. Typical area of analysis was 100 μm × 100 μm.

Prior to SEM and EDS characterization, both the PES and the $LiNO_3$-PES separators were sputter-coated with gold. Lithium metal electrodes for SEM, XPS, and TOF-SIMS analysis were retrieved from Li||Li cells after 100 cycles at a current density of 0.1 mA cm^{-2} and an areal capacity of 0.1 mAh cm^{-2}. Before characterization, lithium metal electrodes were rinsed three times with dimethyl carbonate and then vacuum-dried at 50 °C for 12 h.

Electrochemical measurements

Ion conductivity (σ) is measured by assembling stainless steel (SS)||SS cells and using electrochemical impedance spectroscopy (EIS) under the measurement conditions of 10^{-1}-10^6 Hz with an amplitude of 10 mV, and it can be calculated using the following equation:

$$\sigma=\frac{L}{R\times S} \tag{1}$$

Here, L is the thickness of the electrolyte, R is the electrolyte impedance measured by EIS, and S is the contact area between the electrolyte and the steel electrode.

The σ of the electrolyte at different temperatures within the range of 25-65 °C was tested, and the activation energy of Li^+ transport was calculated using the following equation:

$$\sigma=Ae^{-\frac{E_a}{RT}} \tag{2}$$

Here, A is the pre-exponential constant, E_a is the activation energy, R is the ideal gas constant, and T is the absolute temperature during the test.

The lithium-ion transference number (t_{Li+}) of the electrolyte was calculated using the following equation:

$$t_{Li^+}=\frac{[I_{SS}(\Delta V\text{-}I_0R_0)]}{[I_0(\Delta V\text{-}I_{SS}R_{SS})]} \tag{3}$$

Here, ΔV is the polarization voltage of 10 mV, R_0 and R_{SS} are the interfacial resistances of the Li||Li cells at the initial and steady states, respectively, and I_0 and I_{SS} are the initial current and steady-state current values, respectively.

The electrochemical window of the electrolyte was tested by linear sweep voltammetry (LSV), with a scan rate set at 1 mV s^{-1} and a voltage range of 0-6 V.

Li||NCM811 cells were assembled for electrochemical floating analysis. First, the cells were charged at a constant current to 3.4 V, then the voltage was increased stepwise with 0.1 V increments up to 4.8 V, with a 10-hour constant voltage hold at each voltage plateau.

Cyclic voltammetry (CV) test was conducted on the Cu||Li cells, with a voltage scan range from 2.5 to -0.5 V and a scan rate of 10 mV s^{-1}.

The exchange current density of the Li deposition/stripping process was determined by performing LSV on Li||Li cells and fitting the resulting Tafel curves. The scan rate was 2 mV s^{-1}, with a voltage range of -0.25-0.25 V.

The critical current density (CCD) of the electrolyte was measured in the Li||Li cells. By controlling the charge and discharge duration to 0.5 h each time, the current density was gradually increased from 0.1 to 10 mA cm^{-2} in steps of 0.2 mA cm^{-2}.

The rate performance test of Li||Li cells was based on a fixed charge/discharge areal capacity of 1 mAh cm^{-2}, completed by changing the current density.

The coulombic efficiency (CE) was measured employing the Aurbach method, which is calculated using the following formula:

$$\mathrm{CE}=\left(\frac{nQ_C+Q_S}{nQ_C+Q_T}\right)\times 100\% \qquad (4)$$

Here, n is the number of cycles, Q_T is the initial total Li plating areal capacity (5 mAh cm^{-2}), Q_C is the fixed cycling areal capacity (0.5 mAh cm^{-2}), and Q_S is the areal capacity retention measured during the final Li stripping process. The stripping process was terminated at a cutoff voltage of 1 V.

The charge and discharge voltage range of LFP||Li cells was 2.5-4.0 V; the charge and discharge voltage range of NCM811||Li cells was 2.5-4.3 V. The high-loading cathodes used for assembling the pouch cell measure 4.3 × 5.6 cm^2.

The negative/positive (N/P) ratio is calculated according to the following equation:

$$\frac{N}{P}=\frac{q_{NE}m_{NE}}{q_{PE}m_{PE}} \qquad (5)$$

Here, q_{NE} is the negative gram capacity, m_{NE} is the negative active material mass, q_{PE} is the positive gram capacity, m_{PE} positive active material mass.

Computational methods

Density functional theory (DFT) calculation was performed using Gaussian 16 and GaussView 5.0 software with the B3LYP/6-311++G(d,p) basis set for geometric optimization and energy level calculations of various molecular systems. The binding energy was calculated using the following equation:

$$E_b=E_{total}-\sum E_{frag} \qquad (6)$$

Where E_b is the binding energy, E_{total} is the total energy of the bound system, and $\sum E_{frag}$ is the sum of the energies of all isolated fragments.

Molecular dynamics (MD) simulations were performed using GROMACS package with cubic periodic boundary conditions. The atomistic force field parameters for all are described by the OPLS-AA. The equations for the motion of all atoms were integrated using a classic Verlet leapfrog integration algorithm with a time step of 1.0 fs. A cutoff radius of 1.4 nm was set for short-range van der Waals interactions and real-space electrostatic interactions. The particle-mesh Ewald (PME) summation method with an interpolation order of 4 and a Fourier grid spacing of 0.12 nm was employed to handle long-range electrostatic interactions in reciprocal space. In all three directions, periodic boundary conditions were imposed. Leapfrog algorithm was used to integrate the Newtonian equation of motion. The system was first equilibrated through a 20 ns MD simulation in the NPT ensemble, with pressure isotropically maintained at 1 bar using the Berendsen barostat. Subsequently, a 10 ns production run was conducted in the NPT ensemble for data collection, with pressure isotropically maintained at 1 bar using the Parrinello-Rahman barostat. The temperature was maintained by the V-rescale thermostat at 298.15 K.

Supplementary Figures and Tables

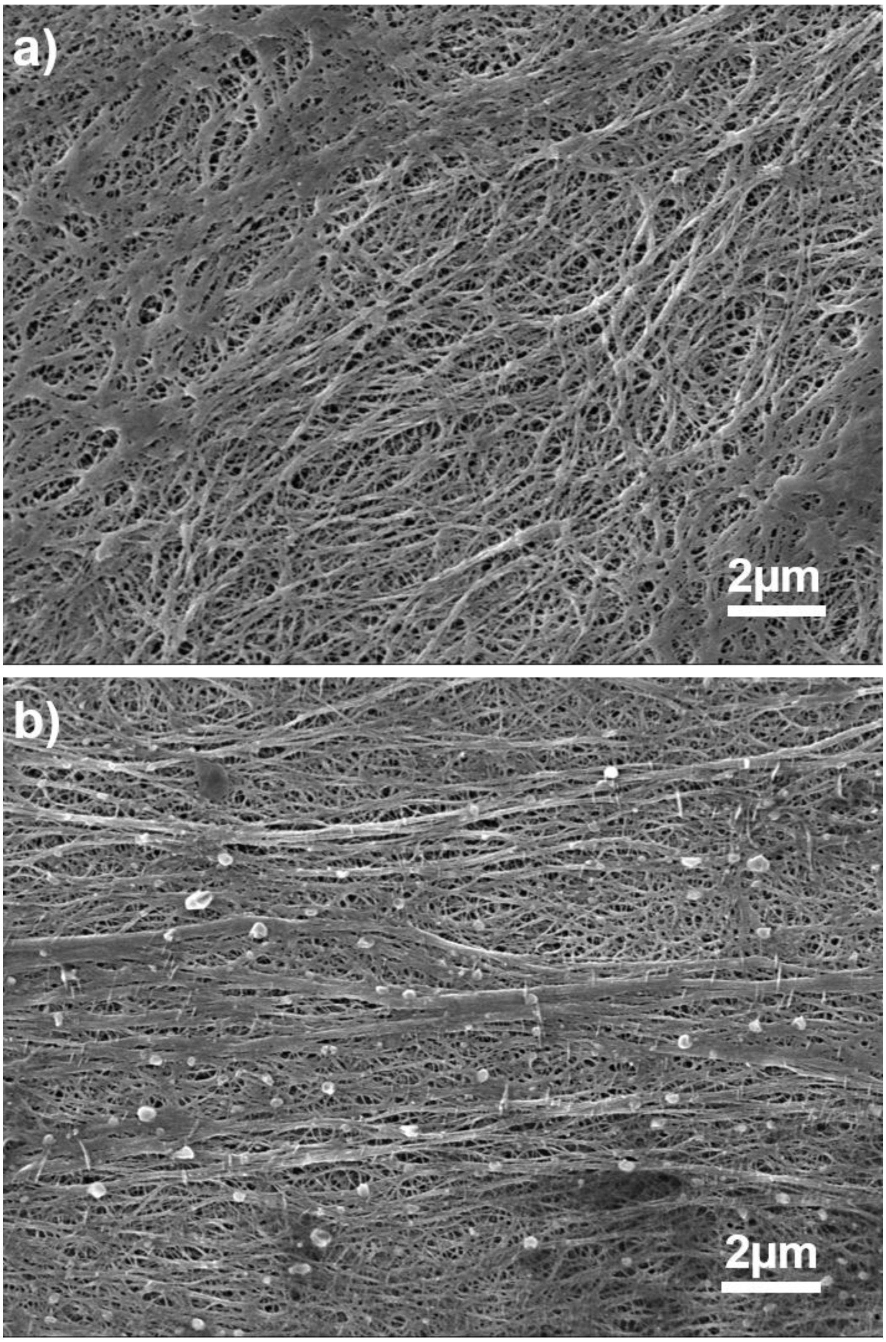

Figure S1. SEM images of PES (a) and $LiNO_3$-PES separators (b).

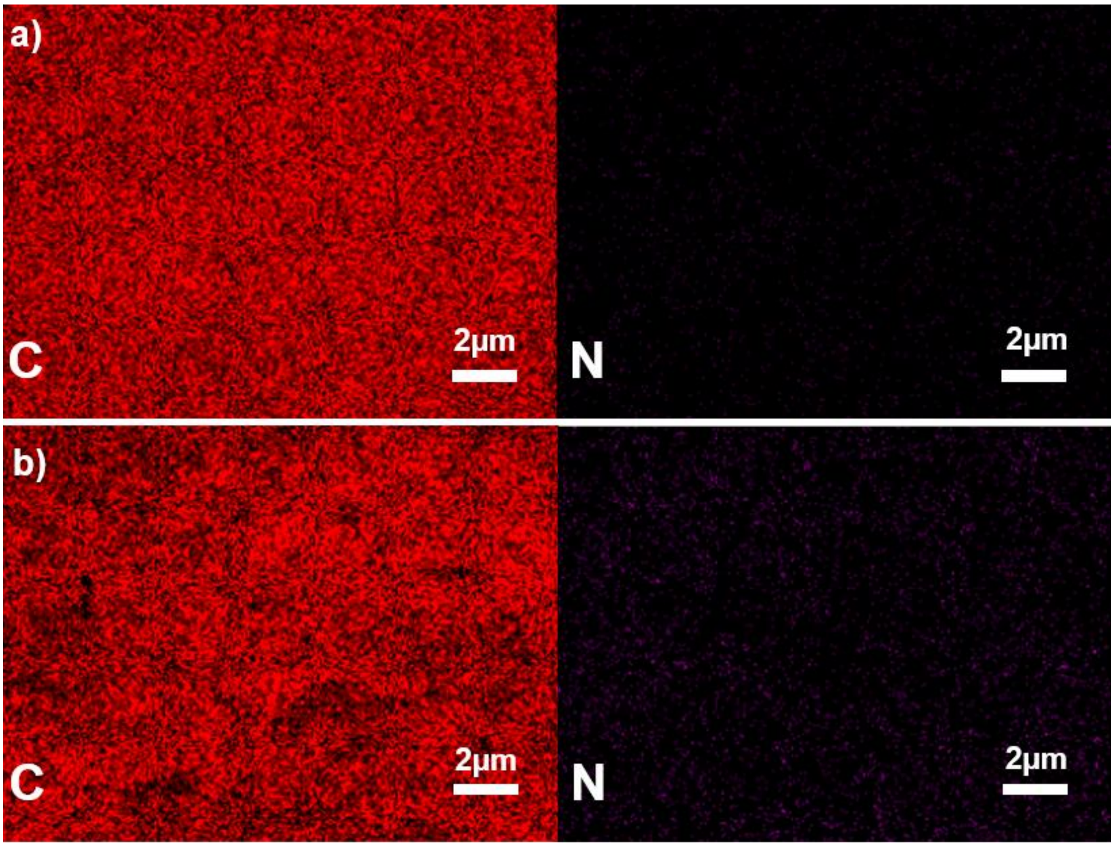

Figure S2. EDS element mappings of C and N elements of PES (a) and $LiNO_3$-PES separators (b).

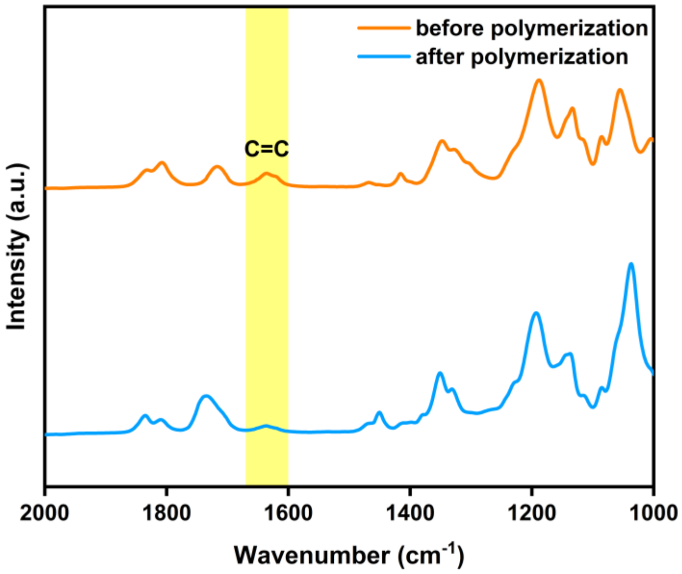

Figure S3. FTIR spectra of the solution of the GPE-FN precursor before and after polymerization.

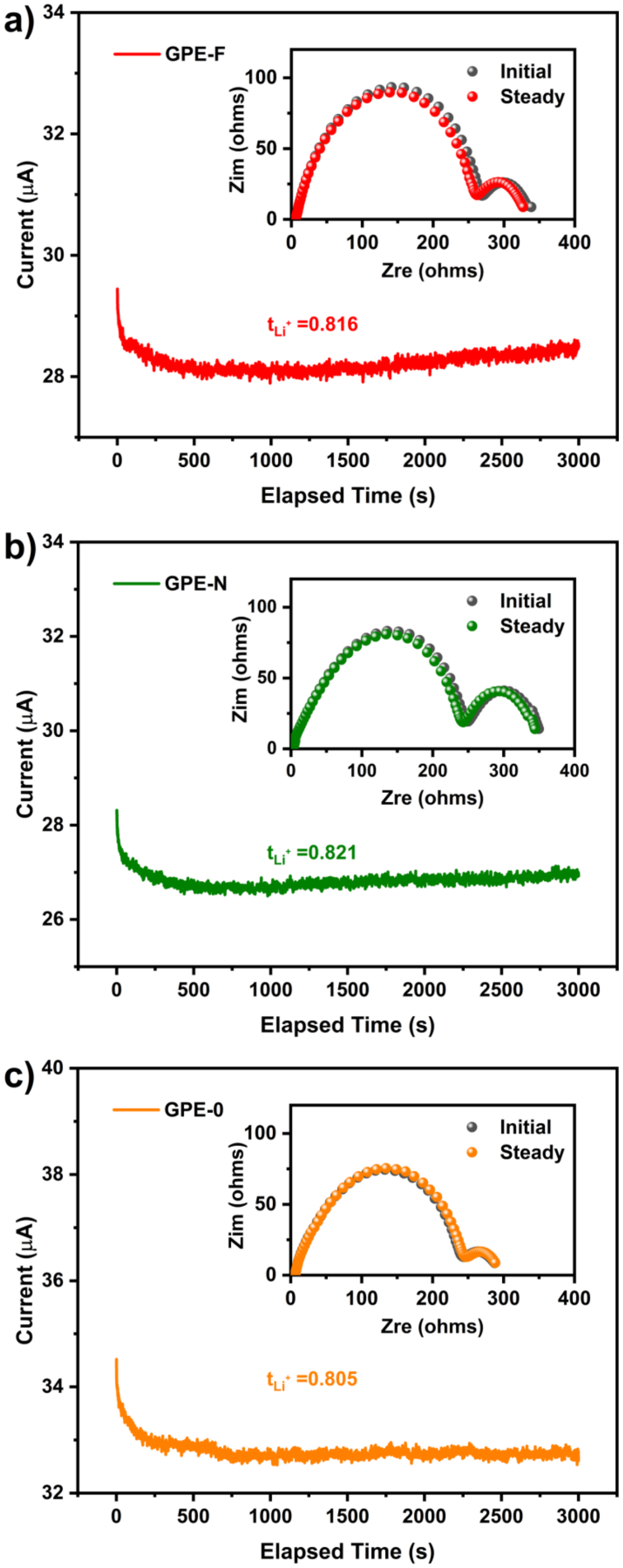

Figure S4. The chronoamperometry profiles of the Li|GPE-F|Li (a), Li|GPE-N|Li (b) and Li|GPE-0|Li (c) cells (inset: the EIS before and after polarization).

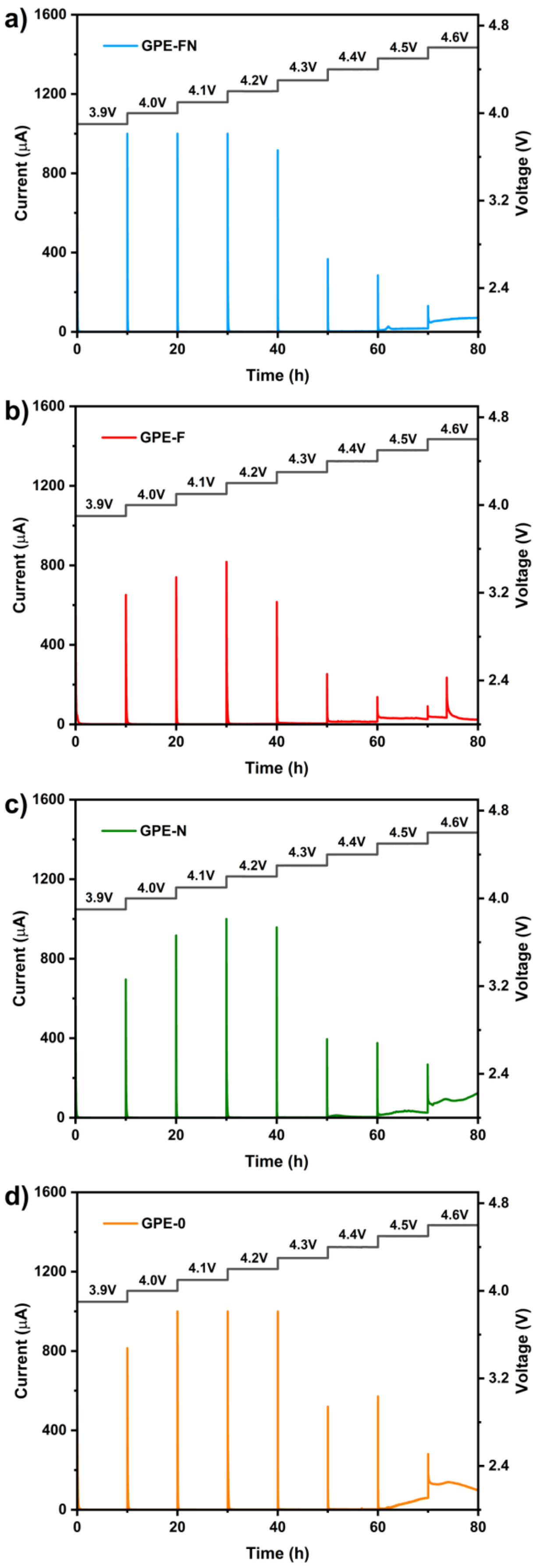

Figure S5. Electrochemical floating analysis of GPE-FN (a), GPE-F (b), GPE-N (c), and GPE-0 electrolytes (d).

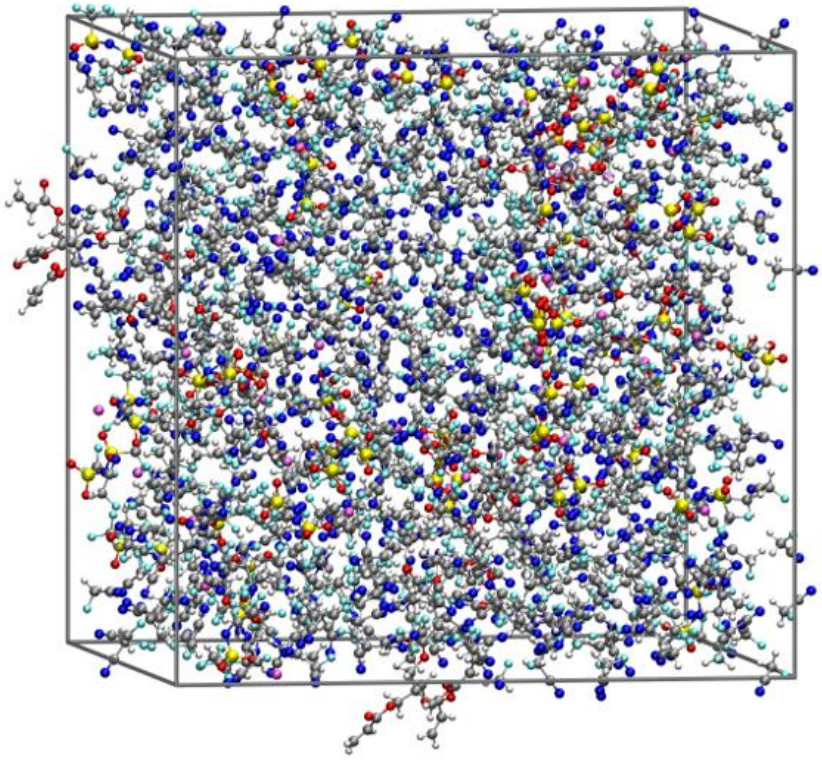

Figure S6. GPE-0 snapshot obtained by MD simulation.

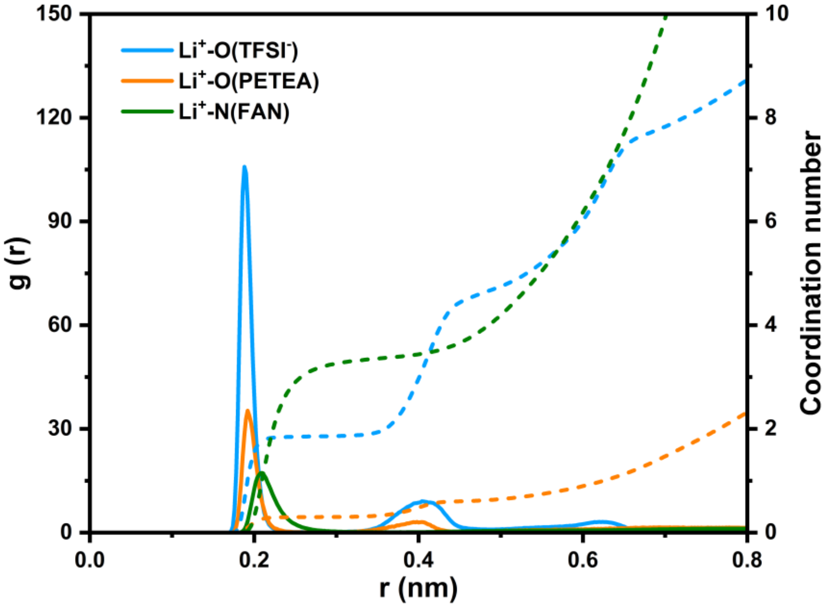

Figure S7. RDF and CN of Li^+ in the GPE-0 electrolyte.

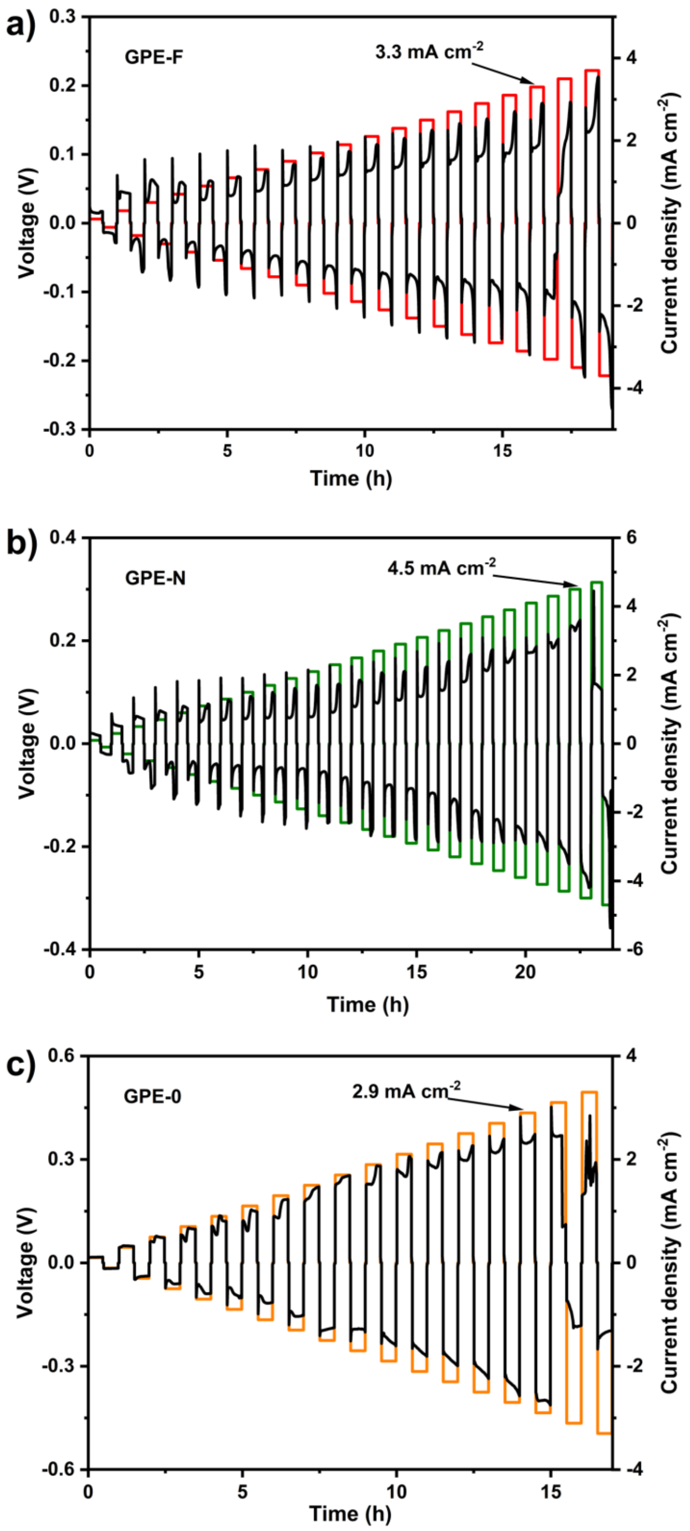

Figure S8. CCD plots of the Li|GPE-F|Li (a), Li|GPE-N|Li (b) and Li|GPE-0|Li (c) cells under step-up current densities with a constant Li plating and stripping time of 0.5 h.

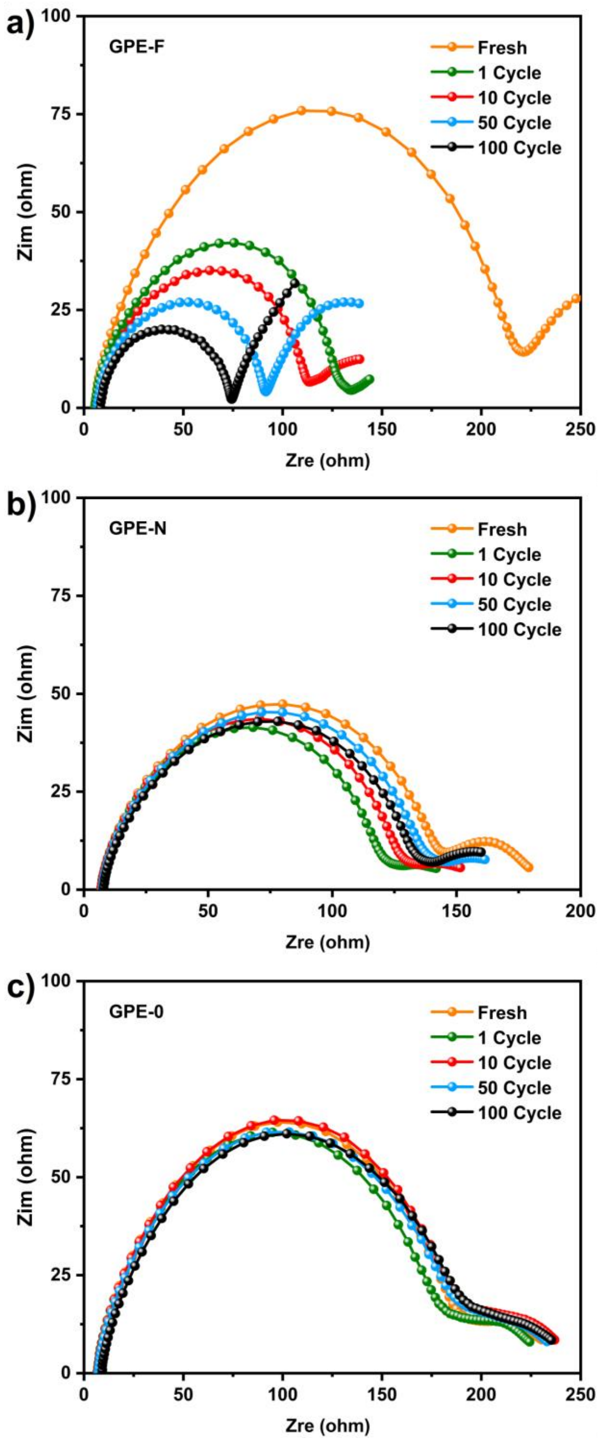

Figure S9. EIS spectra of the Li|GPE-F|Li (a), Li|GPE-N|Li (b), and Li|GPE-0|Li (c) cells before and after various cycle numbers.

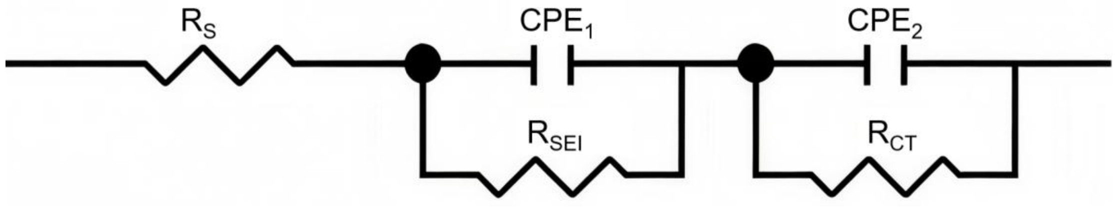

Figure S10. The equivalent circuit for impedance fitting of Li||Li cell with GPE-FN, GPE-F, GPE-N and GPE-0.

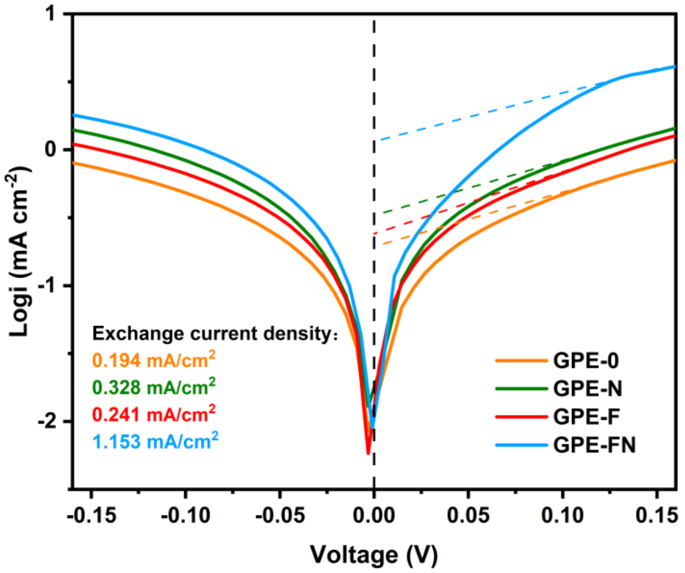

Figure S11. Tafel fitting curves of the Li|GPE-FN|Li, Li|GPE-F|Li, Li|GPE-N|Li, and Li|GPE-0|Li cells.

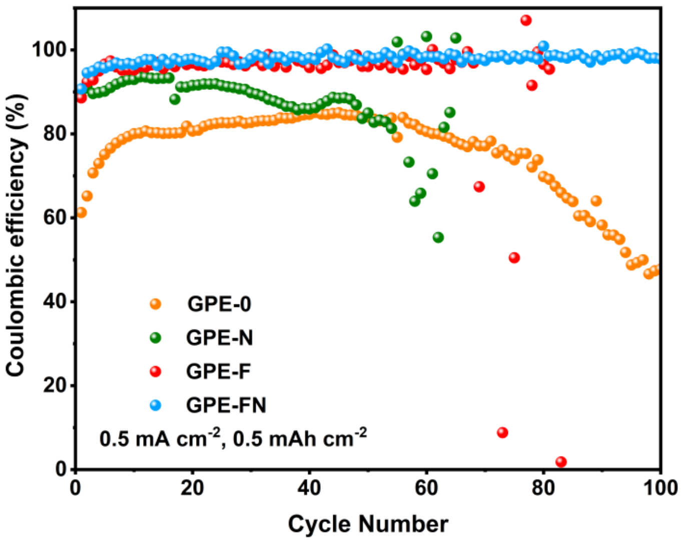

Figure S12. CE of Li|GPE-FN|Cu, Li|GPE-F|Cu, Li|GPE-N|Cu, and Li|GPE-0|Cu cells during the Li plating/stripping on Cu foil at 0.5 mA cm^{-2} and 0.5 mAh cm^{-2}.

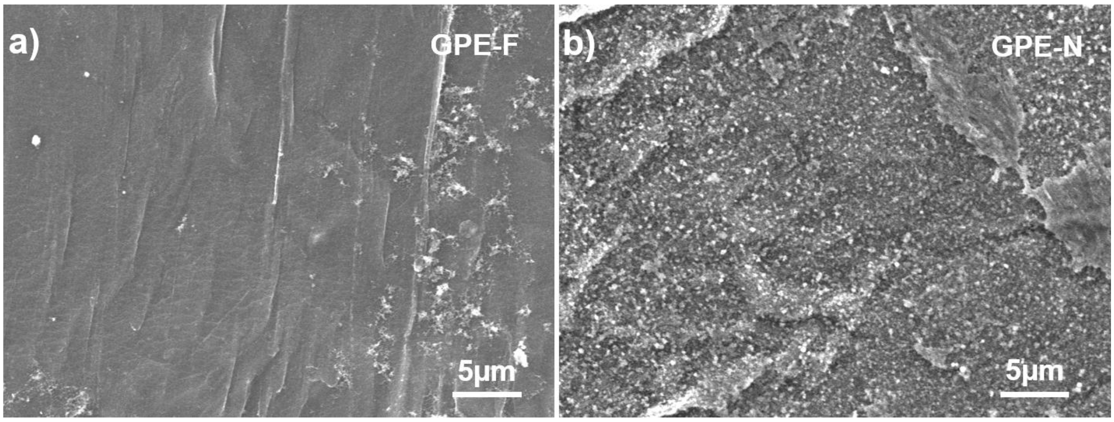

Figure S13. Surface SEM images for lithium metal electrodes of the Li|GPE-F|Li (a) and Li|GPE-N|Li (b) cells after 100 cycles at 0.1 mA cm^{-2} and 0.1 mAh cm^{-2}.

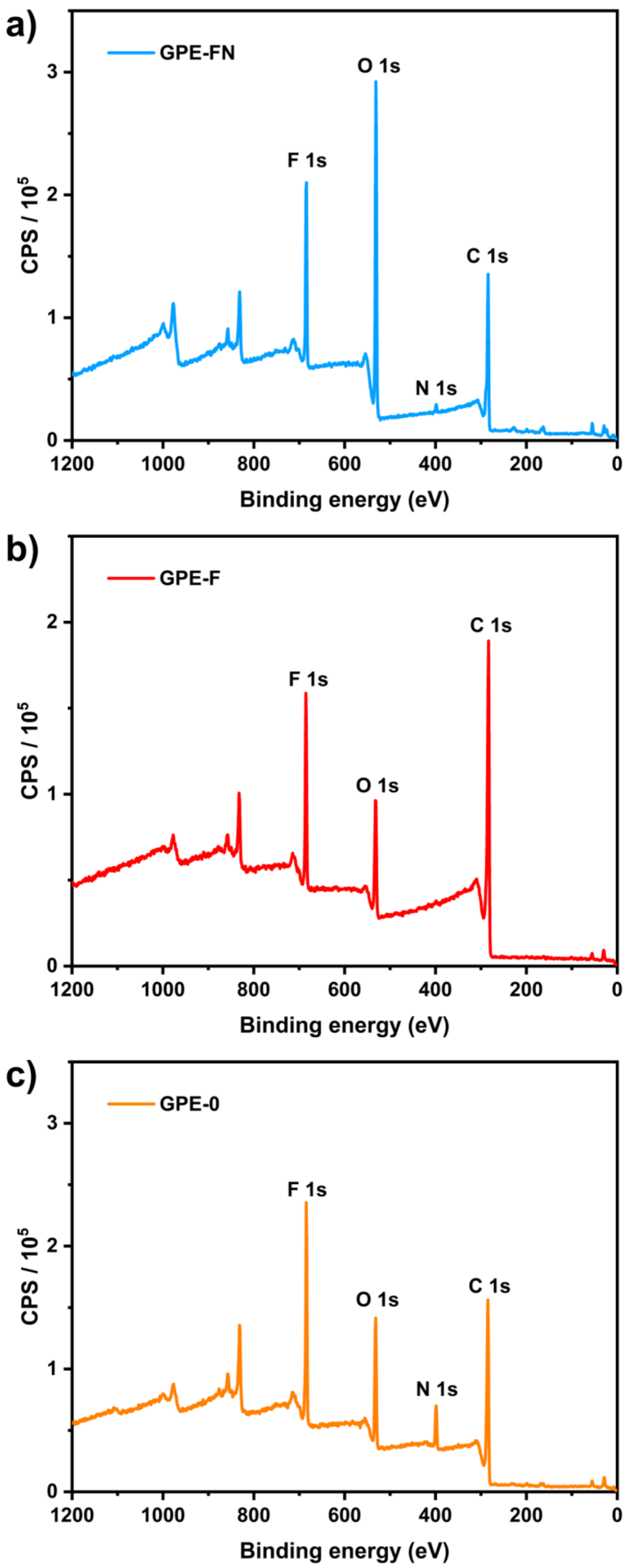

Figure S14. XPS spectra under Ar^{+} etching time of 60 s for the SEI layers of the Li|GPE-FN|Li (a), Li|GPE-F|Li (b), and Li|GPE-0|Li cells (c).

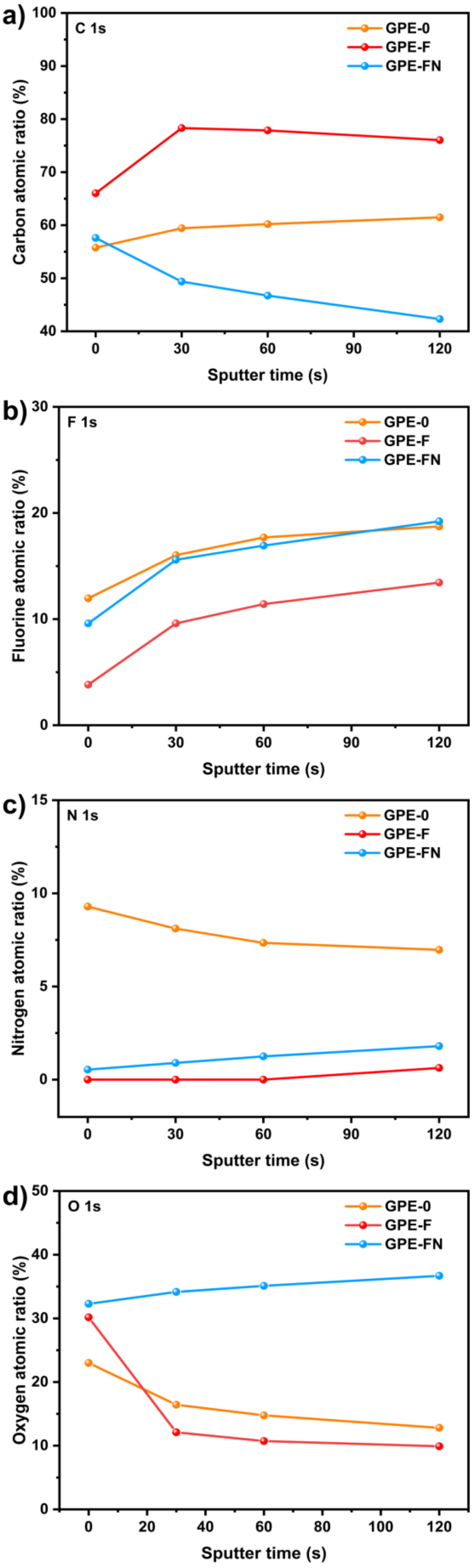

Figure S15. Atomic percentages of C (a), F (b), N (c), and O (d) elements as a function of the sputtering time for the SEI layers of the Li|GPE-FN|Li, Li|GPE-F|Li, and Li|GPE-0|Li cells.

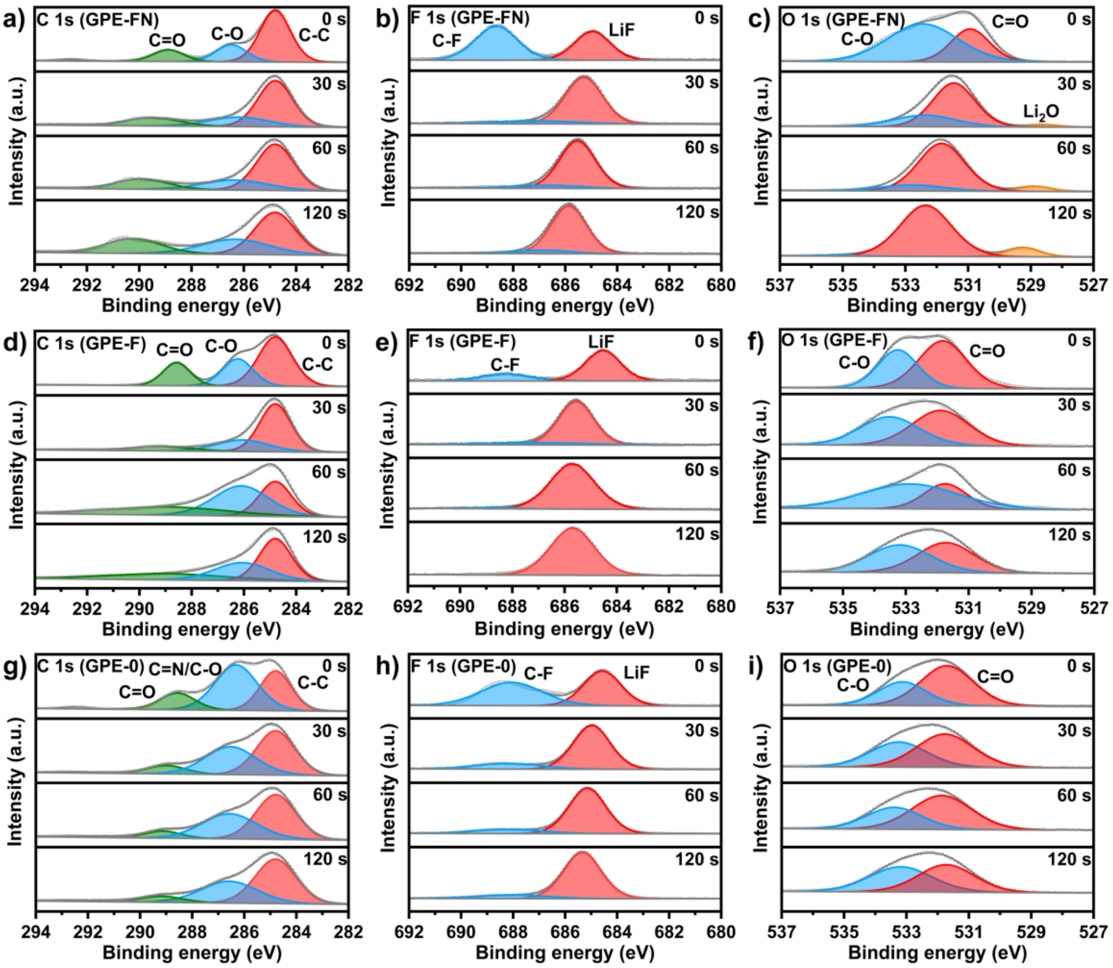

Figure S16. XPS depth profiles of C 1s, F 1s, and O 1s spectra for the SEI layers of the Li|GPE-FN|Li (a-c), Li|GPE-F|Li (d-f), and Li|GPE-0|Li (g-i) cells.

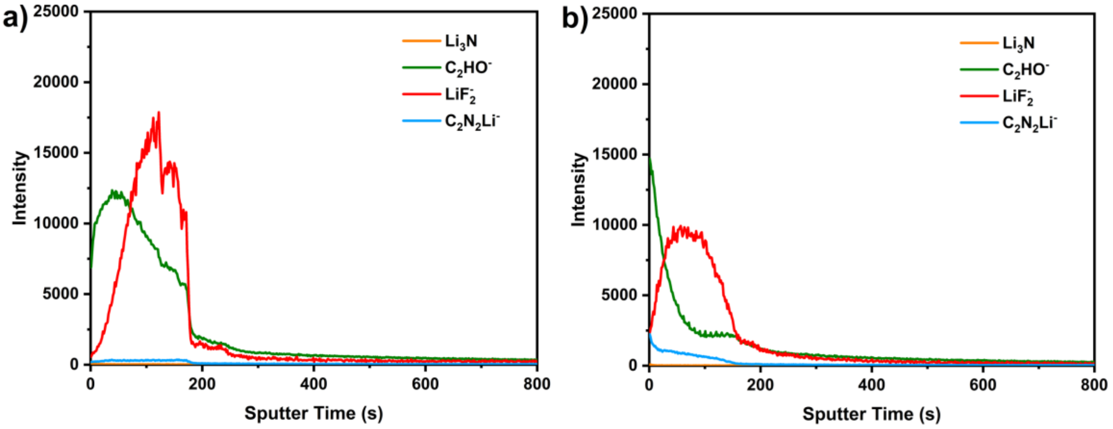

Figure S17. TOF-SIMS depth profiles of the SEI layers of the Li|GPE-FN|Li (a) and Li|GPE-0|Li cells (b).

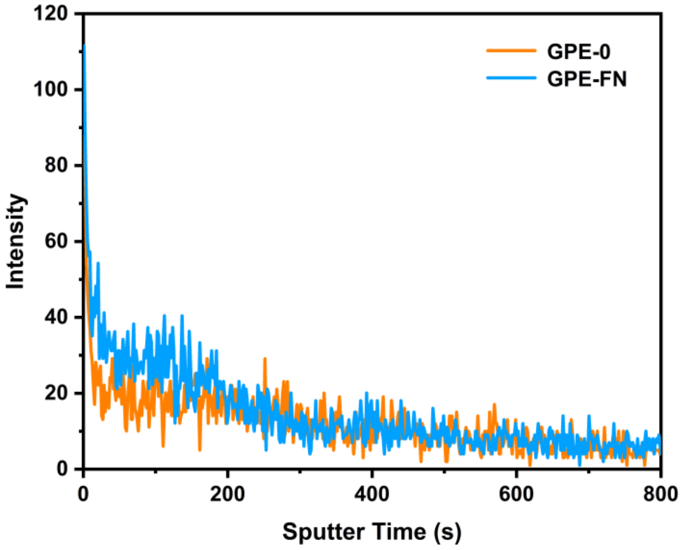

Figure S18. TOF-SIMS depth profiles of Li_3N in the SEI layers of the Li|GPE-FN|Li and Li|GPE-0|Li cells.

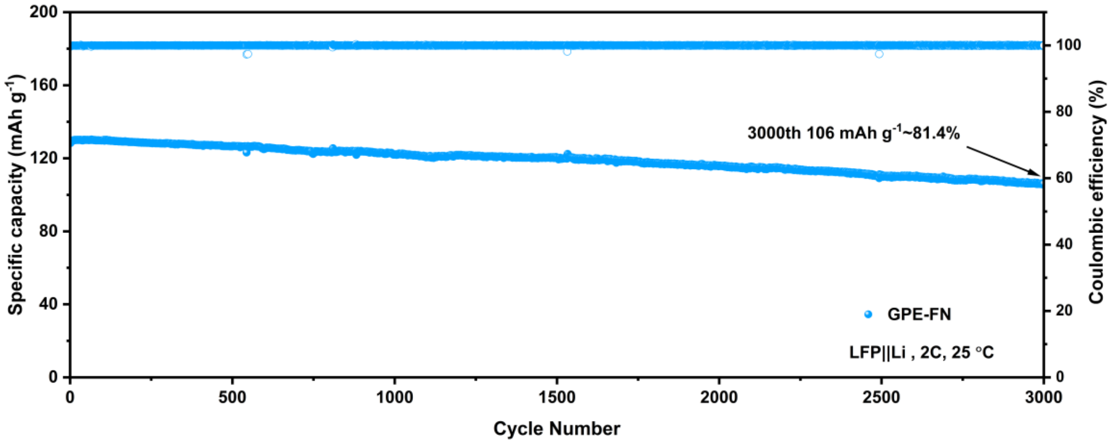

Figure S19. Long-term cycling of the LFP|GPE-FN|Li cell at 2C and 25 °C.

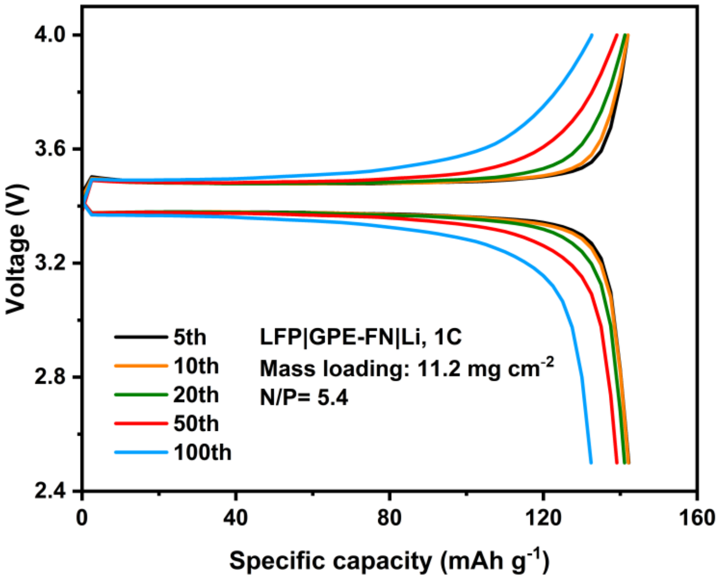

Figure S20. The charging/discharging curves of the LFP|GPE-FN|Li full cell at 1C.

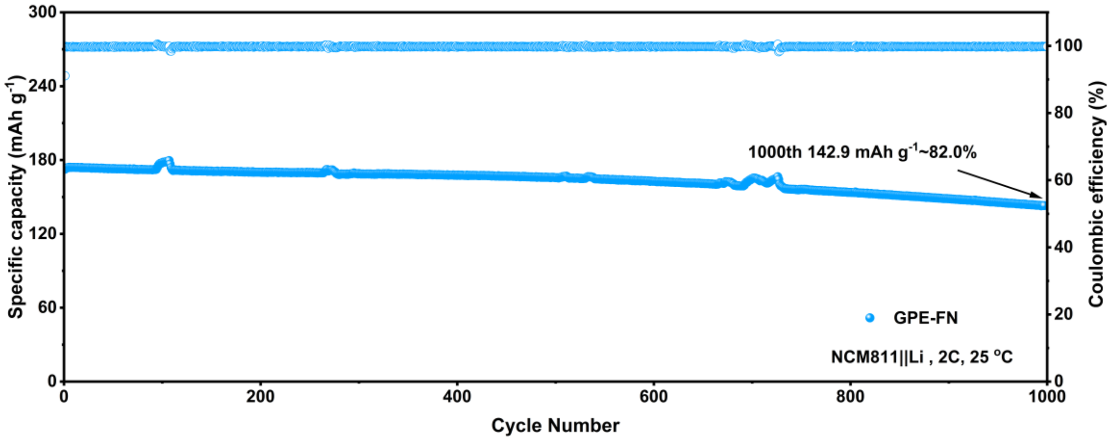

Figure S21. Long-term cycling of the NCM811|GPE-FN|Li cell at 2C and 25 °C.

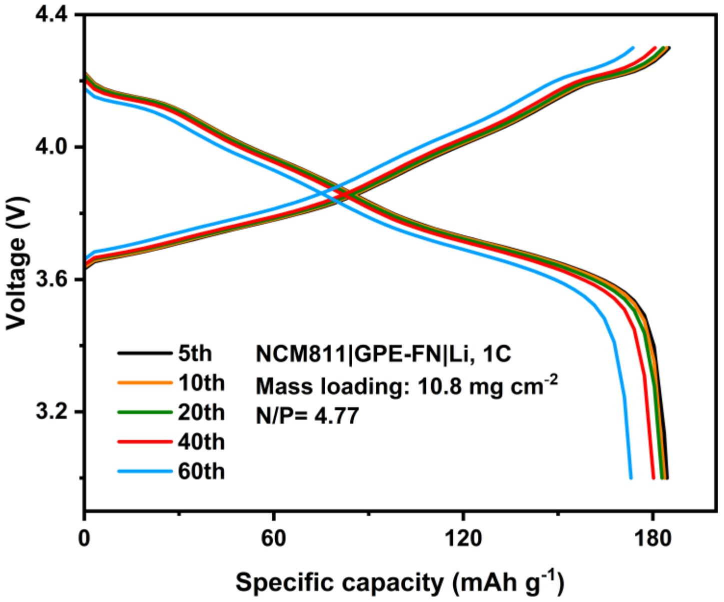

Figure S22. The charging/discharging curves of the NCM811|GPE-FN|Li full cell at 1C.

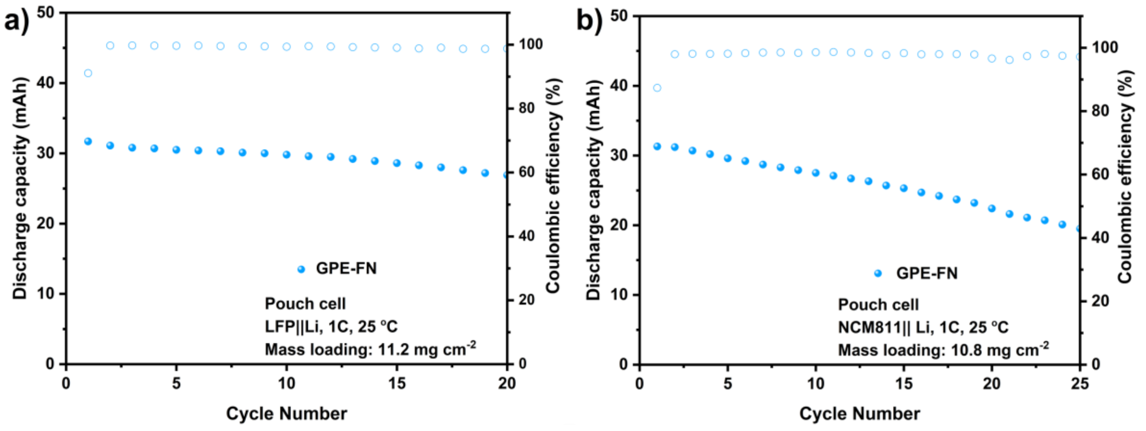

Figure S23. Long-term cycling of the LFP|GPE-FN|Li (a) and NCM811|GPE-FN|Li (b) pouch cells at 1C and 25 °C.

Table S1. Comparison of performance with other gel polymer electrolytes.

Ref	Rate (C)	Specific capacity (mAh g^{-1})	Mass loading (mg cm^{-2})	Cycle number	Capacity retention (%)	Cathode
1	1	137.3	3.6	400	91.8	LFP
2	3	122.7	3.0	340	93.5	LFP
3	5	/	2.0	1300	81.0	LFP
4	2	130.2	2.0	500	82.3	LFP
5	5	118.9	4.0	1000	87.5	LFP
6	2	138.1	2.0	1800	70.5	LFP
7	1	141.1	2.5	500	80.5	LFP
8	1	140.0	4.0	2500	81.7	LFP
9	1	/	2.0	1000	81.1	LFP
10	1	/	3.0	260	87.6	NCM811
11	3	170.0	2.4	665	80.0	NCM811
12	5	/	2.0	300	75.6	NCM811
13	3	136.7	1.2	800	84.7	NCM811
This work	5	121.6	2.0	4100	80.3	LFP
This work	5	163.7	2.0	800	79.6	NCM811

Supplementary references

[1] K. Zeng, Q. Liu, H. Ma, G. Zhao, Q. An, C. Zhang, Y. Yang, M. Sun, Q. Xu, L. Duan, H. Guo, “In situ co-growth LiF-Li_3N rich dual-protective layers enable high interface stability for solid-state lithium-metal batteries” *Energy Storage Materials* **70**, (2024): 103564.

[2] X. Zhang, M. Zhang, J. Wu, X. Hu, B. Fu, Z. Zhang, B. Luo, K. Khan, Z. Fang, Z. Xu, M. Wu, “Lewis acid fluorine-donating additive enables an excellent semi-solid-state electrolyte for ultra-stable lithium metal batteries” *Nano Energy* **115**, (2023): 108700.

[3] Y. He, X. Shan, Y. Li, Z. Li, L. Li, S. Zhao, S. Gao, J. Qu, H. Yang, P.-F. Cao, “In-situ formation of quasi-solid polymer electrolyte for wide-temperature applicable Li-metal batteries” *Energy Storage Materials* **68**, (2024): 103281.

[4] J. Li, J. Chen, X. Xu, Z. Wang, J. Shen, J. Sun, B. Huang, T. Zhao, “Enhanced Interphase Ion Transport via Charge-Rich Space Charge Layers for Ultra-Stable Solid-State Lithium Metal Batteries” *Advanced Energy Materials* **15**, (2025): 2402746.

[5] S. Wang, Y. He, G. Zhang, K. Ma, C. Wang, F. Zhou, Z. Wang, Z. Liu, Z. Lü, X. Huang, Y. Zhang, “Multifunctional Silicon-Based Composite Electrolyte Additive Enhances the Stability of the Lithium Metal Anode/Electrolyte Interface” *Advanced Energy Materials* **14**, (2024): 2401384.

[6] Y. Jiang, S. Zhao, X. Xiao, J. Pi, Y. Wang, N. Yi, L. Zou, Z. Xu, Y. Xiao, X. Ao, G. Ding, W. Zhou, N. Zhou, Z. Xue, “Poly(benzoxazine)-Based Gel Polymer Electrolytes for Lithium Metal Batteries With Ultralong Lifespans” *Angewandte Chemie International Edition* **64**, (2025): e202510997.

[7] Y. Zhang, Z. Li, S. Zhang, J. Li, S. Lei, P. Dong, W. Zeng, J. Wang, X. Chen, D. Li, S. Mu, “High-Elastic Flame-Retardant Polyacrylate-Based Gel Polymer Electrolyte by Dual-Phase Fluorination for Highly Stable Lithium-Metal Batteries” *Nano Letters* **25**, (2025): 4930-4938.

[8] T. Zhang, J. Yu, T. Lin, Y. Song, M. Li, Y. Feng, L. Song, Y. Jiang, W. Xie, Y. Lu, T. Liu, K. Zhang, J. Chen, “Fluorinated Deep Eutectic Gel Electrolytes for Sustainable Lithium Metal Batteries” *Journal of the American Chemical Society* **147**, (2025): 32861-32872.

[9] W. Min, L. Li, M. Wang, S. Ma, H. Feng, W. Wang, H. Ding, T. Cheng, Z. Li, T. Saito, H. Yang, P. Cao, “Mastering the Copolymerization Behavior of Ethyl Cyanoacrylate as Gel Polymer Electrolyte for Lithium-metal Battery Application” *Angewandte Chemie International Edition* **64**, (2025): e202422510.

[10] X. Miao, J. Hong, S. Huang, C. Huang, Y. Liu, M. Liu, Q. Zhang, H. Jin, “In Situ Gel Polymer Electrolyte with Rapid Li^+ Transport Channels and Anchored Anion Sites for High-Current-Density Lithium-Ion Batteries” *Advanced Functional Materials* **35**, (2025): 2411751.

[11] Y. Lu, Y. Liu, S. Zhang, Y. Wu, H. Cheng, Y. Lu, “Toward practical lithium metal batteries via a solvation structure regulation strategy in in situ polymerized fluorinated gel polymer electrolytes” *Energy & Environmental Science* **18**, (2025): 9512-9523.

[12] H. Yang, J. Yan, S. Gao, X. Chen, Y. Wang, H. Huo, C. Fu, C. Du, P. Zuo, “An asymmetric functional gel polymer electrolyte enables superior interfacial compatibility for wide temperature lithium metal batteries” *Energy & Environmental Science* **18**, (2025): 9854-9864.

[13] W. Xu, L. Zhou, S. Lu, J. He, Y. Xu, L. Tian, “Fluorine-free gel polymer electrolyte for lithium oxide-rich solid electrolyte interphase and stable Li metal batteries” *Nature Communications* **16**, (2025): 9308.
